# Measuring job frustration in Omani healthcare workers: development and psychometric validation of the OJFQ

**DOI:** 10.1186/s40359-026-04762-5

**Published:** 2026-05-19

**Authors:** Asiya M. Al Zadjali, Yoke Yong Chen, Keng Sheng Chew, Rekaya V. Balang, Hamida H. Al Harthi, Md Mizanur Rahman, Rachel L. J. Chiew

**Affiliations:** 1 Faculty of Medicine and Health Sciences, Universiti Malaysia Sarawak, Kota Samarahan, Sarawak Malaysia; 2 Mental Health Nursing Program Coordinator, Higher Institute of Health Specialties, Muscat, Oman

**Keywords:** Job frustration, Healthcare workers, Omani job frustration questionnaire, Workplace well-being, Cultural sensitivity scale

## Abstract

**Background:**

The western existing scales to measure employee burnout are often critiqued in relation to cultural sensitivity and early stage job frustration experience while being implemented in non-western settings as evidenced within the limitations of the local study findings. Therefore, since Omani society values social stability while being integrated with employment, there is a demanding necessity for developing a culture-sensitive scale to measure job frustration among healthcare workers (HCWs).

**Objective:**

This study aimed to develop and psychometrically validate the Omani Job Frustration Questionnaire (OJFQ), a culturally grounded instrument designed to assess early-stage occupational frustration among healthcare workers in Oman.

**Methods:**

The development of the scale was considered following a sequential mixed-methods approach. Set of six focus group discussions (FGDs) with 42 nurses in the mental health setting steered initial item generation guided by conceptual frameworks from the MARS model, and CBI. Expert reviews involving 16 professionals from Oman and Malaysia were conducted to refine the item pool ending up with a 45-item OJFQ initially. The newly developed scale was surveyed on 139 participants working in a mental health setting, primarily involving nursing professionals, getting a response rate of 99.3%. The data analysis was conducted in presenting details of descriptive statistics, Cronbach’s alpha, exploratory factor analysis (EFA), item analysis and confirmatory factor analysis (CFA) to assess scale validity.

**Results:**

The final scale included 37-item across five constructs after conducting EFA: (1) management and role clarity, (2) emotional regulation and professional coping strategies, (3) career development and engagement, (4) workplace bureaucracy and social challenges, and (5) workplace resources and stability. The scale demonstrated acceptable internal consistency (Cronbach’s α = 0.774 for the final 37-item version), and all constructs were significantly correlated with the total score. CFA showed an acceptable chi-square ratio but suboptimal fit across most indices, providing preliminary support for the proposed factor structure. Both EFA and CFA were conducted on the same sample. In addition, there was no significant correlation (*r* = 0.032, *p* = 0.709) was found with the CBI, suggesting that the scale may capture a related but distinct construct.

**Conclusion:**

The OJFQ represents a culturally tailored instrument with preliminary psychometric support for assessing job frustration among healthcare workers with initial validation in a mental health nursing context in Oman. Its development may support early identification of occupational strain within culturally specific healthcare environments. Further refinement across diverse healthcare settings and populations are warranted.

**Supplementary Information:**

The online version contains supplementary material available at 10.1186/s40359-026-04762-5.

## Introduction

Existing Western-developed scales have been critiqued for limited cultural sensitivity when implemented in non-western settings as reflected findings from regional studies. Therefore, since Omani society values social stability while being integrated with employment, there is a need to develop a culture-sensitive scale to measure job frustration among healthcare workers (HCWs).

The most commonly used instrument for assessing the level of burnout is the Maslach Burnout Inventory (MBI), which has been criticized by its limited consideration of multidimensional factors beyond work-related domains [1]. Social and personal life domains are closely interconnected with work-related concerns, particularly within Arab sociocultural contexts [2]. In contrary, CBI had considered personal-related concerns as contributing factors to burnout, however, it is not culture-sensitive specially in non-western settings [2]. Elbarazi et al [2] found that there are significant discrepancies in scoring of burnout among HCWs within Arab countries using the same scale which was MBI. This highlights the need for a job frustration assessment tool that addresses limitations in existing instruments, which primarily assess the earlier manifestations of occupational distress, i.e., job frustration, before progression to burnout and ending up with mental health issues. The existing instruments may be less sensitive in detecting psychological strain that may lead to burnout on long run. Therefore, assessing job frustration provides a more proactive and preventive approach to understanding occupational well-being, particularly in high-demand healthcare settings.

In Oman, people value social life and financial security which are achieved through being employed. However, there is absence of locally developed and validated scale which is considered culturally sensitive to measure job frustration constructs in Oman that is distinct and measurable construct that may contribute to early identification of occupational strain and inform timely interventions before progression to burnout. Existing scales creates a gap in the accurate and relevant assessment guidance towards effective management of HCWs job frustration [2, 3].

Therefore, given the sociocultural importance of employment in reflecting social structure, job frustration arises due to personal and societal related constructs requiring a more culture-context instrument. Job frustration is conceptualized in this study as a multidimensional construct reflecting perceived barriers and unmet expectations in the workplace that impede professional functioning, distinct from, yet conceptually related to burnout, job stress, and job dissatisfaction, representing an early stage affective-intellectual response to occupational barriers and unmet expectations [4–6]. Unlike burnout, which is typically characterized by emotional exhaustion, depersonalization, and reduced personal accomplishment, job frustration may emerge earlier within the occupational stress continuum and it could be the first sign of stress at work before more serious burnout symptoms appear [7, 8]. In contrast to job stress, which generally reflects physiological and psychological strain responses arising from work demands [9], job dissatisfaction represents employees’ evaluative attitudes and perceptions toward their work environment and job experiences [10]. Emotional exhaustion, meanwhile, constitutes a core dimension of burnout associated with energy depletion and fatigue, whereas job frustration may occur before exhaustion becomes clinically or occupationally pronounced.

Healthcare workers' experiences, interpretations, and expressions of occupational distress may be influenced by cultural and contextual factors. Workplace stress responses, emotional expression, coping strategies, and perceptions of organizational support can differ across intercultural contexts [11, 12]. According to Morrison and Milliken [13], employees in collectivist cultures may place a higher value on social stability, professional commitment, and interpersonal harmony than on openly expressing emotional pain or job discontent. As a result, culturally influenced emotional and cognitive experiences that are not always adequately represented by traditional burnout-focused questionnaires created in various organizational and cultural contexts may be a manifestation of job frustration. Cross-cultural healthcare research has also brought attention to similar issues with cultural differences in burnout perception and occupational stress assessment [14].

Despite the fact that job satisfaction and occupational burnout have been extensively researched in healthcare settings, these concepts might not adequately represent culturally rooted experiences of job frustration in the Omani context. Sociocultural norms that emphasize social peace, professional responsibility, collectivism, job stability, and respect for organizational hierarchy have an impact on Omani workplace culture [15, 16]. While maintaining professional functioning and interpersonal harmony, healthcare workers may experience ongoing internal frustration related to organizational inefficiencies, limited autonomy, unclear role expectations, restricted career advancement, or perceived inequities in such environments [17, 18].

Instead of immediately manifesting as burnout or overt job dissatisfaction, these experiences may emerge as an earlier affective-cognitive state characterized by tension, blocked goals, emotional repression, and perceived workplace restrictions [5, 19]. Current tools for measuring burnout, such the Maslach Burnout Inventory (MBI) and the Copenhagen Burnout Inventory (CBI), mainly evaluate more severe signs of workplace stress, such as emotional exhaustion, depersonalization, and disengagement [7, 8]. As a result, these tools might be less sensitive to early-stage, culturally influenced experiences of job frustration that come before burnout [4]. The Omani Job Frustration Questionnaire (OJFQ) was therefore created with the goal of offering a contextually relevant and culturally grounded tool that can detect early signs of occupational strain among Omani healthcare workers.

**Fig. 1:**
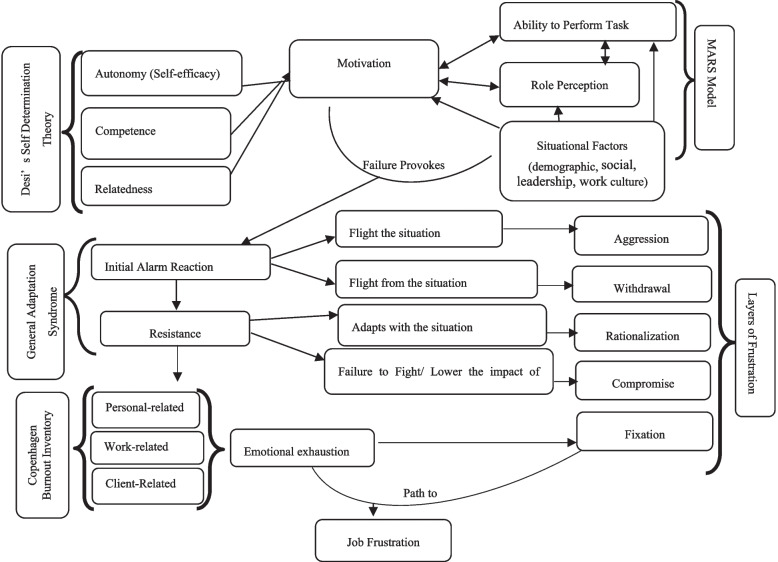
Integrated conceptual framework of development of job frustration

This study aimed to develop and psychometrically validate the Omani Job Frustration Questionnaire (OJFQ), a culturally grounded instrument designed to assess early-stage occupational frustration among healthcare workers in Oman. Grounded in the MARS model [20] and informed by the theoretical conceptualizations derived from the Maslach burnout inventory (MBI) [1], Copenhagen burnout inventory (CBI) and other related theories (Fig. 1) [14, 21, 22], this study developed and validated a new job frustration scale to assess the level of job frustration among HCWs in Oman. This study followed a sequential approach to scale development and validation [3]. The integrated conceptual framework conceptualizes job frustration as an intermediate psychological state within the occupational stress continuum, linking job stressors and long-term burnout (Fig. 1). Although the current study focuses primarily on a mental health nursing setting, the conceptual framework of job frustration may have broader applicability across healthcare professions and warrants further validation in diverse clinical contexts.

## Materials and methods

### Study design and sample

The study sample was drawn exclusively from a mental health care setting and primarily involved nursing professionals, which should be considered when interpreting the generalizability of the findings. The sample size was considered adequate for psychometric evaluation and factor analysis based on established recommendations suggesting participant-to-item ratios ranging from 5:1 to 10:1 for scale development studies [23, 24]. Additional methodological guidance supporting adequate sample sizes for exploratory and confirmatory factor analyses was also considered [3].

A total of six FGDs were conducted in the qualitative phase to explore participants’’ experiences and perspectives with varying groups of nurses i.e. general nurses and nursing leaders involving 42 nurses from an estimated workforce of 400 nurses in one of the mental hospitals in Oman. Data collection continued until thematic saturation was achieved. These nurses were purposively recruited based on their professional roles and availability after getting their written consent to participate. The FGDs were guided by the theoretical dimensions of the MARS model (motivation, ability, role perception and situational factors). Conceptual domains derived from the MBI (emotional exhaustion, depersonalization, personal accomplishment) and CBI (personal-related, work-related and client-related factors) were additionally used to inform the generation of potential Omani Job Frustration Questionnaire (OJFQ) items.

In the quantitative phase, purposive sampling was used to recruit mental health nurses working within mental health setting in Muscat. This population was selected due to their frequent exposure to emotionally demanding clinical environments, organizational pressures, and complex interpersonal interactions, making them particularly relevant for exploring experiences related to occupational frustration. Inclusion criteria included registered nurses with direct clinical experience in mental health settings and a minimum duration of professional work experience within the healthcare system.

### Measures

The initial scale items were generated from through the FGD findings with consideration of their demographic characteristics including age, gender, work experience and field of practice. The initial scale construction was started with literature review on relevant literature. The time period considered for data collection through FGDs was from February to May 2023. The purposively selected participants were invited to join 90-min interviews. Set of six FGDs were conducted considering 42 total participants including 3 sessions for each group of nurses (general nurses and nursing leaders). The interviews were audio-recorded, transcribed and later analysed using Otter.ai transcription software which resulting in the generation of 45 preliminary items.

The Content Validity Index (CVI) was evaluated using a 4-point rating scale assessing both item relevance and recommendation status separately (where 1 = not relevant and not recommended, 2 = somewhat relevant and somewhat recommended, 3 = relevant and recommended and 4 = highly relevant and highly recommended). CVI reflects expert agreement regarding specific item relevance with scoring of 3 and 4. Based on preliminary exploratory scale-development consideration, the CVI value of 0.49 and above was considered as per Yusoff et al. [25] for inclusion with an inter-rater agreement value evaluation through modified kappa requiring to score above 0.40. Therefore, out of 16 experts (12 Omani and 4 Malaysian) divided into two phases where 8 experts allocated in each phase in this study. This number of experts is considered methodologically adequate for content validation procedure [26]. The selected experts are highly qualified and experienced in research who were 7 nurses (5 lecturers, 2 administrators), 8 doctors (2 emergency, 3 psychiatry, 1 epidemiology, 1 nephrology, 1 administration) and 1 psychologist among whom 4 were Malaysian (2 emergency doctors, a psychiatrist and a nurse lecturer) who evaluated the items’ relevance and suitability in assessing job frustration of HCWs. Following the two phases of expert review, the 45 items were set on 7-point Likert scale (1 = strongly disagree, 2 = disagree, 3 = somewhat disagree, 4 = neutral, 5 = somewhat agree, 6 = agree, 7 = strongly agree) which was named as Omani Job Frustration Questionnaire (OJFQ). The 45-items considered for reverse scoring of 18 items on basis of where higher scores reflected greater level of job frustration (Table 1). For reliability and validity check, the scale was pilot tested on 140 participants on basis of 3:1 ratio representing 1 item for 3 participants.

**Table 1:** List of items for reverse scoring

Item Number	Statement
N 1	My emotional well-being significantly improves when a manager demonstrates empathy
N 2	My understanding of the stressors enhances my affective state
N 3	I can effectively reduce stress by openly discussing my concerns with my managers
N 6	My performance improves with the presence of supervisors
N 8	My level of commitment to task is enhanced when I involve in decision-making
N 10	I feel less frustrated when the workload is distributed fairly in my workplace
N 16	My current working environment is safe for me to practice new skills
N 17	I am having a supportive relationship with my leader that reduce my job frustration and improves my performance
N 18	I am getting positive reinforcement and encouragement at work like everyone else
N 36	My ability to tolerate crises greatly influences my stress levels at work
N 37	My resilience at work improves when I can effectively understand and manage emotions
N 38	My ability to recognize emotions in myself and others improves my coping skills
N 39	My faith or religious beliefs provide me with strength to cope with work-related stress
N 40	Having strong support from my family helps to reduce my job dissatisfaction
N 42	I receive constructive feedback that help improving my skills, performance and learning that lead to less job frustration
N 43	I am actively participating in own learning process to reduce my job frustration
N 44	My training materials are directly applicable to my daily tasks that reduce my job frustration
N 45	I feel that staying updated with the latest trends in healthcare helps me manage my job frustration

### Instrument scoring

Several items in the OJFQ were negatively worded to reduce response bias. These items were reverse scored prior to analysis so that higher scores consistently reflected higher levels of job frustration. For a 7-point Likert scale, reverse scoring was performed as follows: 1 = 7, 2 = 6, 3 = 5, 4 = 4, 5 = 3, 6 = 2, and 7 = 1. The reverse-coded items included total of 18 items which are: N1, N2, N3, N6, N8, N10, N16, N17, N18, N36, N37, N38, N39, N40, N42, N43, N44 and N45 (Table 1).

### Analysis

Qualitative data were analyzed using thematic analysis. The data obtained for this study were first transcribed and translated into English where necessary as some participants used Arabic terminologies to ensure convenience and understanding by other authors. It was followed by an iterative and inductive coding process using NVivo 14 software through a six-step process developed by Braun and Clarke [27] (Table 2), i.e., familiarizing with the data, data familiarization, initial coding, theme development, theme review, theme definition, and report generation, while ensuring accuracy by referring to recorded data. The MARS Model was used as a sensitizing theoretical framework through the six phases of thematic analysis was considered throughout the analysis of the data aiding in identification of the themes [28] (Table 2).

**Table 2:** Incorporation of MARS model with six-step process of Braun and Clarke [27] of thematic analysis

Phase	Direction	Actual Action
Phase 1	Getting to Know the Data	Reading through the qualitative data obtained from FGDs to familiarize with it. Clear out instances in which participants bring up elements of the MARS model, such as: situational factors (e.g., poor work environment), role perception (e.g., unclear job expectations), ability (e.g., feeling underqualified), or motivation (e.g., lack of rewards)
Phase 2	Making the first codes	As to begin to code the data, the codes are created that reflect the MARS factors. For example, codes could include terms like "Undervalued reward system," "role clarity," "insufficient resources," or "negative work conditions."
Phase 3	Searching for themes	The codes would be grouped into more general categories surfacing the MARS framework. A collection of codes pertaining to "lack of motivation" can, for example, create a topic regarding employee disengagement. Similarly, a theme about inadequate training and development may be formed by codes pertaining to capacity (e.g., role perception and clarity)
Phase 4	Examining the themes	The themes would be examined and improved to make sure they appropriately depict the facts. They can be combined or reinterpreted themes of motivation (like irritation over unclear roles) if they appear to overlap with role perception themes (like confusion about job tasks) to ensure that they clearly relate to the MARS model
Phase 5	Themes definition and naming	Each theme would explicitly be outlined after being decided on them. "Frustration Due to Role Ambiguity," for instance, may be a theme that closely relates to role perception according to the MARS model. The theoretical foundation of each theme is ensured by connecting them to MARS
Phase 6	Putting together the report	Writing the analysis would include making links between the MARS model and the themes that were found. It would describe how issues like low motivation or a lack of skills lead to job dissatisfaction and provide data examples to show how each MARS factor appears in workers' experiences

Interview transcripts were repeatedly reviewed to achieve data familiarization prior to initial coding. Codes were subsequently grouped into broader categories based on conceptual similarities, and themes were refined through continuous comparison across participant responses. The analytic process focused on identifying recurring experiences related to workplace barriers, emotional strain, coping responses, and organizational challenges associated with job frustration. To enhance methodological rigor, ongoing review and refinement of themes were conducted throughout the analysis process. Data collection continued until thematic saturation was reached from subsequent interviews.

The MARS model [20] offered a framework for comprehending the various reasons why people become frustrated at work, and theme analysis enabled the identification and interpretation, arrange, and analyse the data in a way that emphasizes these reasons. In order to increase the validity of the study and guarantee that the conclusions were logically supported by the literature on organizational behaviour, each stage of the thematic analysis method aided in identifying patterns and themes that can be directly mapped to the MARS variables.

### Data management

During the qualitative phase, data management in terms of processing, storage, organization and secure handling overall was maintained. This ensure ethical handling of sensitive data for the purpose of the study.

The audio recordings as well as verbatim transcriptions of the focused group interviews were stored in password protected folders in password-protected institutional computer used solely by the main researcher and shared through encrypted institutional drive with other co-researchers as a backup copy. The recordings and the verbatim transcriptions were anonymous with unique code for each participant. The stored data are retained for reference throughout the study period. In both phases, the data will be stored with the principal researcher for a period of five years and later will be permanently discarded.

### Trustworthiness and reflexivity

In reference to Lincoln and Guba [29] Model regarding the trustworthiness of the qualitative data representing four criteria which are: credibility, transferability, dependability and confirmability [30]. The rigor of the method utilized is ensured through initial check for accuracy of interview interpretation (credibility), mutual agreement regarding coding of data throughout the data collection process (dependability), reflexive note keeping and peer discussion for agreement over ideas to control bias (confirmability) and clarity of the approach description particularly the context, setting and participants for applicability to different settings (transferability).

Additionally, the researcher as a mental health professional with extensive clinical, academic, and administrative experience acknowledge the potential for interpretive bias. In order to avoid such kind of bias, reflexive note-taking was considered throughout the research study and discussing it with co-researchers, peers, supervisors and experts to ensure the authentic representation of study participants’ voice.

The qualitative findings indicated the multidimensional nature of job frustration from the qualitative phase had indicated multi-dimensional feature of job frustration and adaptive measures among nurses in mental health setting in Oman. These data required to be operationalized to enable statistical assessment, therefore, the generated themes and sub-themes were converted into items for the purpose of initial development of Omani Job Frustration Questionnaire (OJFQ).

### Item generation from qualitative findings

Prior to statistical analysis, negatively worded items were reverse scored to ensure consistent directionality across the scale. Using SPSS version 27 and Jamovi version 2.6 for Microsoft office, exploratory factor analysis (EFA) was conducted to extract number of factors and retain valid items being the most appropriate statistical approach for exploring underlying structure of scale [31]. While considering the principal component analysis with varimax rotation, initial extraction suggested a larger factor solution,however, scree plot inspection and conceptual interpretability supported retention of a five-factor model [32]. This is because the first factor loading showed 13 factors with multiple items demonstrated weak or cross-factor loadings. Scree plotting of the factor was considered at eigenvalue greater than 1 to support the fixed number of factors. Item retention decisions were based on factor loadings (≥ 0.40), conceptual relevance, and contribution to overall construct representation. The loadings above 0.3 was considered for inclusion as it is indicated that lower loadings from 0.3 to 0.4 are slightly acceptable [25]. The minimum ratio was considered due to the practical constraints in accessing a larger independent sample for data collection may cause limited response. Therefore, 139 responses were received from nurses working in mental health setting through online questionnaire of 45-items being rated at 7-point Likert scale, where “1 = strongly disagree, 2 = disagree, 3 = somewhat disagree, 4 = neutral, 5 = somewhat agree, 6 = agree and 7 = strongly agree”. The Kaiser–Meyer–Olkin (KMO) and Barlett’s test for sampling adequacy is performed to checked the overall significance value of the findings in terms of correlation matrix [31]. The reliability of the scale as well as each construct and their correlation with the overall scale is measured.

The retained items were subsequently evaluated using confirmatory factor analysis (CFA). Jamovi version 2.6 software was used to evaluate construct validity through model fit indices of five factor model of the developed scale. As well as, path analysis was conducted through structural equation modelling diagram. Due to sample size constraints, both exploratory and confirmatory factor analyses were conducted on the same dataset. Therefore, the confirmatory findings should be interpreted as preliminary rather than definitive validation. To examine construct distinctiveness, Pearson’s correlation analysis was conducted between the total OJFQ score and the CBI scores.

Prior ethical approval was obtained from the University Malaysia Sarawak (UNIMAS) Medical Research Ethics Committee (No.: FME/22/65) and the Research Approval Committee at MOH in Oman (No. Moh/CSR/22/25809). Informed consent was obtained from the purposively selected sample who agreed to participate in the study.

## Results

### Demographic characteristics of the participants

The study was conducted in a mental health setting in Oman where the questionnaire was distributed to 140 participants with 139 participants completed responses (response rate of 99.3%). The detailed statistical analysis is shown in Table 3 summarizing participant demographic characteristics including age, gender, work experience, and field of practice.

**Table 3:** Demographic characteristics of the participants (*N* = 139)

Item	N (%)	Mean		SD
Age (years)	-	33.18	+	6.207
Work experience (years)	-	9.72	+	5.803
Gender				
Male	67 (47.5%)			
Female	73 (51.8%)			
Field of practice				
Clinical	61 (43.3%)			
Leadership	77 (54.6%)			

### Phase 1: qualitative phase

#### Item generation and analysis

The initial item pool was generated through qualitative exploration of job frustration among nurses in mental health setting. Data were collected using six focus group discussions and followed by thematic analysis procedures. Emerging themes informed the development of a comprehensive set of items reflecting key factors of job frustration, including workplace challenges, psychological responses, social influences and management-related factors. These items formed the preliminary version of the OJFQ.

Item analysis represented that the average total score was 179.1 (SD = 22.1) from all participants. The items with the highest scores were Q5 “I am stressed when there is lack of teamwork” with average total score of 5.7 (SD = 1.2), and Q32 “working on shift duty (shift work) is affecting my social life” with average total score of 5.7 (SD = 1.4). Whereas the items with lowest score were Q39 “My faith or religious beliefs provide me with strength to cope with work-related stress” with average total score of 2.00 (SD = 1.00), and Q40 “having strong support from my family helps to reduce my job dissatisfaction” with average total score of 2(SD = 1). The skewness and kurtosis represented 0.205 and 0.407 respectively suggesting normality. Correlation analysis between individual items and the total scale score indicated that several items demonstrated non-significant correlations. However, they were included due to the good factor loading and conceptual contribution to the construct with other significant scorings (Table 4).

**Table 4:** Item analysis of OJFQ (*N* = 139)

Items	Mean	SD	Skewness		Kurtosis		t	95% CI		Correlation with total score
			Statistic	Std. Error	Statistic	Std. Error		Lower	Upper	
Total Score of Scale	179.0504	22.09608	-.222-	.206	.322	.408	95.536	175.3446	182.7562	1
Q1	2.17	1.258	1.387	.205	2.114	.407	20.428	1.96	2.38	.168*
Q2	2.09	1.042	1.297	.205	1.158	.407	23.680	1.91	2.26	.238**
Q3	2.71	1.407	1.055	.205	.810	.407	22.773	2.47	2.94	.280**
Q4	5.33	1.476	−1.020-	.205	.489	.407	24.711	5.08	5.58	.411**
Q5	5.70	1.216	−1.525-	.205	2.808	.407	55.481	5.50	5.90	.107
Q6	3.16	1.576	.966	.205	.018	.407	23.757	2.90	3.43	.198*
Q7	4.83	1.799	-.942-	.205	-.241-	.407	31.757	4.53	5.13	.136
Q8	2.06	1.051	1.431	.205	3.084	.407	23.159	1.88	2.23	.110
Q9	5.62	1.354	−1.293-	.205	1.299	.407	49.114	5.40	5.85	.182*
Q10	2.46	1.305	1.110	.205	.645	.407	22.277	2.24	2.68	-.036-
Q11	5.13	1.559	-.980-	.205	.162	.407	38.932	4.87	5.39	.322**
Q12	5.36	1.425	−1.075-	.205	.841	.407	44.528	5.13	5.60	.450**
Q13	4.86	1.532	-.669-	.205	-.263-	.407	37.558	4.61	5.12	.583**
Q14	4.92	1.532	-.622-	.205	-.279-	.407	38.016	4.67	5.18	.513**
Q15	5.24	1.478	-.984-	.205	.355	.407	41.965	5.00	5.49	.267**
Q16	3.02	1.620	.963	.205	-.041-	.407	22.064	2.75	3.29	.490**
Q17	2.38	1.135	1.160	.205	1.135	.407	24.805	2.19	2.57	.502**
Q18	2.86	1.447	.773	.205	-.296-	.407	23.359	2.62	3.10	.656**
Q19	4.44	1.855	-.391-	.205	−1.141-	.407	28.290	4.13	4.75	.649**
Q20	4.64	1.734	-.450-	.205	-.853-	.407	31.673	4.35	4.93	.495**
Q21	4.58	1.650	-.398-	.205	-.869-	.407	32.741	4.31	4.86	.257**
Q22	4.28	1.957	-.252-	.205	−1.260-	.407	25.874	3.95	4.61	.519**
Q23	5.10	1.416	-.581-	.205	-.206	.407	42.624	4.86	5.34	.460**
Q24	5.06	1.425	-.568-	.205	-.187-	.407	42.038	4.83	5.30	.364**
Q25	4.89	1.455	-.638-	.205	-.218-	.407	39.737	4.64	5.13	.333**
Q26	4.79	1.492	-.482-	.205	-.476	.407	37.944	4.54	5.04	.392**
Q27	4.26	1.922	-.229-	.205	−1.306-	.407	26.258	3.94	4.59	.320**
Q28	5.05	1.538	-.832-	.205	-.004-	.407	38.859	4.79	5.31	.225**
Q29	4.71	1.557	-.486-	.205	-.535-	.407	35.770	4.45	4.97	-.135-
Q30	4.91	1.586	-.679-	.205	-.443-	.407	36.601	4.64	5.17	.310**
Q31	5.26	1.506	-.858-	.205	-.131-	.407	41.362	5.01	5.52	.295**
Q32	5.67	1.375	−1.122-	.205	.917	.407	48.796	5.44	5.90	.268**
Q33	5.48	1.486	−1.132-	.205	.515	.407	43.623	5.23	5.73	.384**
Q34	4.89	1.539	-.718	.205	-.468-	.407	37.608	4.64	5.15	.299**
Q35	4.23	1.715	-.223-	.205	−1.005-	.407	29.179	3.94	4.52	.266**
Q36	2.65	1.175	1.172	.205	1.234	.407	26.689	2.45	2.85	.582**
Q37	2.21	.973	.983	.205	1.119	.407	26.929	2.05	2.38	.583**
Q38	2.31	1.150	1.361	.205	2.070	.407	23.740	2.11	2.50	.515**
Q39	1.96	1.024	1.473	.205	2.602	.407	22.615	1.79	2.13	.636**
Q40	1.96	1.045	1.507	.205	2.599	.407	22.164	1.78	2.13	.027
Q41	5.04	1.416	-.680-	.205	-.104-	.407	42.070	4.80	5.27	.119
Q42	2.94	1.358	.974	.205	.617	.407	25.575	2.71	3.16	.034
Q43	2.70	1.262	1.173	.205	1.295	.407	25.313	2.49	2.91	.087
Q44	2.81	1.267	.787	.205	-.034-	.407	26.280	2.60	3.03	.288**
Q45	2.34	1.174	1.197	.205	1.662	.407	23.615	2.15	2.54	.285**

^**^Correlation is significant at the 0.01 level (2-tailed)

^*^Correlation is significant at the 0.05 level (2-tailed)

**Fig. 2:**
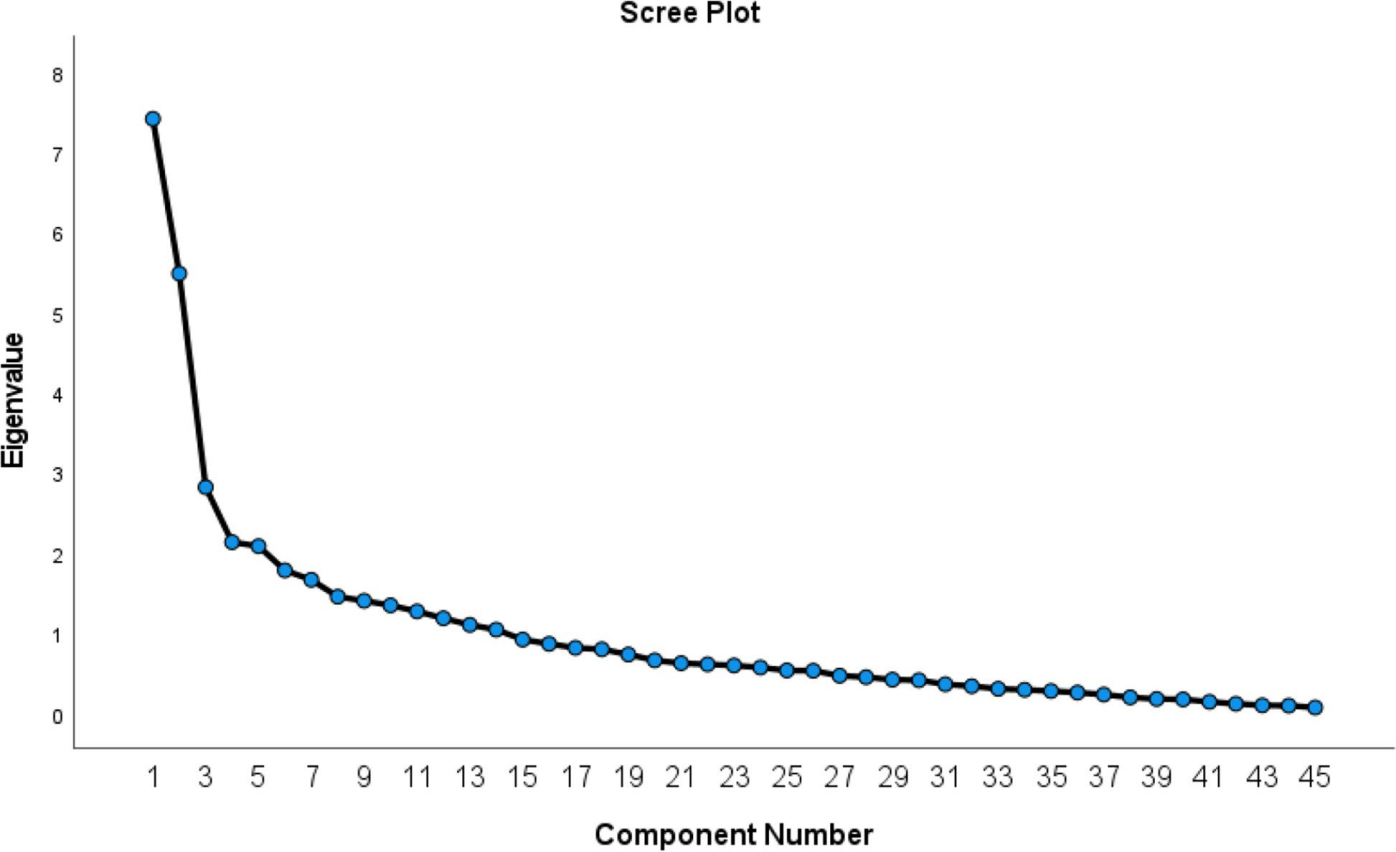
Scree plot performed on 5-factors with Eigenvalue greater than 1

In reference to the factor analysis and loading, the factors identified and named as (1) “management and role clarity”, (2) “emotional regulation and professional coping strategies”, (3) “career development and engagement”, (4) “workplace bureaucracy and social challenges” and (5) “Workplace resources and stability”. However, the elbow at scree plot (Fig. 2) shows a sharp decline across the first three factors, and followed by gradual stabilization of the eigenvalues until construct 5 suggesting the most significant factors. Therefore, iterative factor analyses were subsequently conducted was considered using a fixed five-factor solution where the elbow of scree plot appeared with adjusted constructs. Factor retention decisions were based on multiple criteria, including eigenvalues greater than 1, scree plot inspection, factor interpretability, and conceptual alignment with the underlying theoretical framework.

The review considered some changes in the items. To illustrate, within the management and role clarity factor, item 21 was merged with item 22 as *“I have fear of blame while making decision or writing incident reports”* and item 29 was merged with item 31 as *“I need to put more effort to balance between professional and personal life”*. Additionally, items 7 *“I prefer to adapt to the current stressful environment rather than start from zero in another setting of healthcare practice”* and 26 *“There is a lack of autonomy, empowerment, and independence in management at first line level”* were removed due to poor conceptual alignment with the extracted factors. Items 3 *“I can effectively reduce stress by openly discussing my concerns with my managers”*, 9 *“I get frustrated when colleagues mix up their job duties with their personal relationships with leaders”* 33*“I am physically and mentally insecure by working in mental healthcare field”* and 40 *“Having strong support from my family helps to reduce my job dissatisfaction”* were removed due to low factor loadings across all factors.

### Phase 2: content validity

#### Expert review

Content validity was assessed through expert evaluation of the initial item pool over two phases. A panel of sixteen experts from both Oman and Malaysia with relevant experience in mental health, healthcare practice and research methodology were invited. Experts rated each item for relevance and recommendation status using a 4-point Likert scale (1 = not relevant to 4 = highly relevant) as well additional suggested items to be included (Table 5). Item-level content validity index (I-CVI) was calculated as the proportion of experts rating the item as either 3 or 4. The probability of chance agreement (Pc) and modified kappa (K) were also computed to adjust for chance agreement.

**Table 5:** Item-level content validity index (I-CVI) and modified Kappa for OJFQ (merged phase 1 and phase 2 expert panel review)

Item	N	A	CVI	Pc	Modified Kappa (K)	Interpretation of modified kappa (K)
Factor 1: Management and role clarity						
1 I get frustrated when colleagues mix up their job duties with their personal relationships with leaders	15	9	0.2	0.00030	0.198	Poor
2 I feel lack of confidence due to poorly defined job descriptions and task allocations among different members of the healthcare multidisciplinary team	15	14	0.87	0.00046	0.870	Excellent
3 there is extreme interference from top management in leaders’ tasks	15	12	0.6	0.0139	0.594	Good
4 there is a waste of effort in succession planning since the selection of new healthcare leaders comes solely from top authorities	15	11	0.47	0.04166	0.447	Fair
5 My concerns are acknowledged by the top management, but no substantial actions are taken to address them	15	13	0.73	0.04166	0.718	Good
6 it is hard to change basic policies in the hospital to adapt to the dynamicity of technological advances	16	12	0.5	0.02777	0.486	Fair
7 My current working environment is safe for me to practice new skills (suggested)						
8. I am having a supportive relationship with my leader that reduce my job frustration and improves my performance (suggested)						
9. I receive constructive feedback that help improving my skills, performance and learning that lead to less job frustration (suggested)						
10. I am actively participating in own learning process to reduce my job frustration (suggested)						
11. My training materials are directly applicable to my daily tasks that reduce my job frustration (suggested)						
12. I feel that staying updated with the latest trends in healthcare helps me manage my job frustration (suggested)						
13 My emotional well-being significantly improves when a manager demonstrates empathy	16	14	0.75	0.00183	0.750	Excellent
14 level of commitment to task is enhanced when I involve in decision-making	15	15	1.00	0.00003	1.00	Excellent
15. My resilience at work improves when I can effectively understand and manage emotions	16	15	0.88	0.00024	0.880	Excellent
16. I feel less frustrated when the workload is distributed fairly in my workplace	15	15	1.00	0.00003	1.00	Excellent
17. My frustration caused by unclear/lack of policies and procedures	15	13	0.73	0.04166	0.718	Good
18. I feel overwhelmed doing additional administrative responsibilities under unhelpful management policies	15	13	0.73	0.04166	0.718	Good
19. I am frustrated by the demanding administration works	15	13	0.73	0.04166	0.718	Good
20. I am often blamed when a clinical-related adverse incident occurs	15	13	0.73	0.04166	0.718	Good
21. If I accept extra work responsibilities, I will be blamed for the extra work not being done as required	16	13	0.63	0.00854	0.627	Good
22. I have fear of blame while making decision or writing incident reports	15	13	0.73	0.04166	0.718	Good
23. There is gender stereotype in healthcare related fields that stressed me up	16	15	0.88	0.00024	0.880	Excellent
24. I need to put more efforts to balance between professional and personal life	16	13	0.63	0.00854	0.627	Good
25. Working on shift duties is affecting my social life	16	14	0.75	0.00183	0.750	Excellent
26. I can effectively reduce stress by openly discussing my concerns with my managers	16	15	0.88	0.00024	0.880	Excellent
27. My ability to tolerate crises greatly influences my stress levels at work	14	13	0.86	0.00085	0.860	Excellent
28. My ability to recognize emotions in myself and others improves my coping skills	15	12	0.6	0.0139	0.594	Fair
29. My faith or religious beliefs provide me with strength to cope with work-related stress (suggested)						
30. Having strong support from my family helps to reduce my job dissatisfaction (Suggested)						
31. I feel demotivated when coming to work with all requests or opinions being rejected	16	16	1.00	0.000015	1.00	Excellent
32. I as a healthcare worker prefer to remain silent at work	14	12	0.71	0.00555	0.708	Good
33. I feel that congested offices with a lack of privacy are not conducive to achieving tasks comfortably	15	14	0.87	0.00046	0.870	Excellent
34. I encounter physical risks while working in the mental health field	16	14	0.75	0.00183	0.750	Excellent
35. I am at risk of developing mental health challenges as a result of my work in this field	16	14	0.75	0.00183	0.750	Excellent
36. Worries about losing my job make me less satisfied with my current position	17	13	0.53	0.018157	0.521	Fair
37. I am stressed when there is lack of teamwork	16	13	0.63	0.00854	0.627	Good

◦ The formula for modified kappa statistic (κ) = (CVI– pc)/(1– pc), where pc represents probability of a chance occurrence

◦ P_c_ is the probability of chance of occurrence. The formula for pc is: N!/[A! *(N-A)!] *0.5^N^ where N = the number of judges, A = the number agreeing on good relevance and recommended

◦ Evaluation criteria for modified kappa (κ): κ = poor (< 0.40), κ = fair (0.40–0.59), κ = good (0.60–0.74) and κ = excellent (> 0.74)

◦ CVI should be 0.88 and above to establish validity with a *p* < 0.05, CVI < 0.49 has to be rejected

Although several items demonstrated lower I-CVI and modified kappa values, their retention was guided by a combined evaluation of statistical performance, factor loadings, and theoretical relevance. Notably, a number of items with lower content validity indices showed strong and stable loadings in the factor analysis, supporting their contribution to the underlying construct.

This was particularly evident in domains related to work environment, leadership dynamics, and organizational culture, where expert agreement was more variable, but items reflected context-specific and sensitive aspects of workplace frustration identified in the qualitative phase. Such discrepancies may reflect differences in expert interpretation rather than true lack of relevance.

Given the exploratory nature of this study and the aim to preserve the conceptual breadth of job frustration, these items were retained to ensure comprehensive construct representation. This approach is consistent with recommendations in early-stage scale development, where theoretical and contextual significance may justify retention alongside statistical criteria.

### Phase 3: quantitative analysis

#### Exploratory factor analysis and reliability

The overall sampling adequacy was measured using KMO and Bartlett’s Test of sampling adequacy scoring 0.694 which showed significance value of < 0.001. This supports the suitability of the data for factor analysis. The reliability and validity statistical analysis was performed. Internal consistency reliability was assessed using Cronbach’s Alpha and composite reliability (CR) indices. Construct validity was evaluated using multiple model fit indices (Table 8). The extracted factors in this phase were further assessed in relation to factor loadings > 0.30 orthogonal varimax rotation axis, eigenvalue > 1.0 and scree plot was used to determine the numbers of factors to be considered.

As a result, five factors were extracted through principal component analysis (PCA) (Table 5). PCA with varimax rotation was used as an initial exploratory approach to reduce items and identify a parsimonious structure, as recommended for early-stage measurement tool development and data reduction purposes [23, 24]. The first factor contained ten items (Q14, Q19-Q21, Q23-Q25, Q27, Q32 and Q41) representing management and role clarity. The second factor contained eight items (Q1-Q2, Q5, Q8, Q10 and Q37-Q39) representing emotional regulation and professional coping strategies. The third factor contained eight items (Q6, Q16-Q18 and Q42-Q45) representing career development and engagement. The fourth factor contained seven items (Q4, Q11-Q13, Q15 and Q30-Q31) representing workplace bureaucracy and social challenges. The fifth and last factor contained four items (Q28 and Q34-Q36) representing workplace resources and stability (Table 6). The level of cumulative explained variance of these five factors is 45.3% which is considered acceptable for psychological scale development indicating good representation of the factors within the dataset.

**Table 6:** Rotated factors for principle component analysis of OJFQ

Items	Factor Loading				
	**I**	**II**	**III**	**IV**	**V**
Factor 1: Management and role clarity					
OJFQ 24 There is a waste of effort in succession planning since the selection of new healthcare leaders comes solely from top authorities	0.745				
OJFQ 25 My concerns are acknowledged by the top management, but no substantial actions are taken to address them	0.713				
OJFQ 23 There is extreme interference from top management in leaders’ tasks	0.702				
OJFQ 41 It is hard to change basic policies in the hospital to adapt to the dynamicity of technological advances	0.578				
OJFQ 19 I am often blamed when a clinical-related adverse incident occurs	0.570				
OJFQ 32 Working on shift duties is affecting my social life	0.521				
OJFQ 21 Shift supervisors are reluctant to make clinical decisions without consulting their immediate manager	0.493				
OJFQ 14 There is unclear delineation of roles among different members of the healthcare multidisciplinary team	0.477				
OJFQ 20 If I accept extra work responsibilities, I will be blamed for the extra work not being done as required	0.476				
OJFQ 27 I as a healthcare worker prefer to remain silent at work	0.474				
Factor 2: Emotional regulation and coping responses					
OJFQ 8 My level of commitment to task is enhanced when I involve in decision-making		0.751			
OJFQ 37 My resilience at work improves when I can effectively understand and manage emotions		0.712			
OJFQ 1 My emotional well-being significantly improves when a manager demonstrates empathy		0.684			
OJFQ 5 I am stressed when there is lack of teamwork		0.679			
OJFQ 38 My ability to recognize emotions in myself and others improves my coping skills		0.618			
OJFQ 2 My understanding of the stressors enhances my affective state		0.577			
OJFQ 10 I feel less frustrated when the workload is distributed fairly in my workplace		0.510			
OJFQ 39 My faith or religious beliefs provide me with strength to cope with work-related stress		0.400			
Factor 3: Career development and engagement					
OJFQ 44 My training materials are directly applicable to my daily tasks that reduce my job frustration			0.735		
OJFQ 43 I am actively participating in own learning process to reduce my job frustration			0.730		
OJFQ 42 I receive constructive feedback that help improving my skills, performance and learning that lead to less job frustration			0.705		
OJFQ 16 My current working environment is safe for me to practice new skills			0.674		
OJFQ 45 I feel that staying updated with the latest trends in healthcare helps me manage my job frustration			0.631		
OJFQ 17 I am having a supportive relationship with my leader that reduce my job frustration and improves my performance			0.588		
OJFQ 18 I am getting positive reinforcement and encouragement at work like everyone else			0.530		
OJFQ 6 My performance improves with the presence of supervisors			0.503		
Factor 4: Workplace bureaucracy and social challenges					
OJFQ 12 I feel overwhelmed doing additional administrative responsibilities under unhelpful management policies				0.674	
OJFQ 30 There is gender stereotype in healthcare related fields that stressed me up				0.639	
OJFQ 11 My frustration caused by unclear/lack of policies and procedures				0.598	
OJFQ 13 I am frustrated by the demanding administration works				0.547	
OJFQ 31 I need to put more efforts to balance between professional and personal life				0.500	
OJFQ 15 I feel lack of confidence due to poorly defined job descriptions and task allocations among different members of the healthcare multidisciplinary team				0.495	
OJFQ 4 I feel demotivated when coming to work with all requests or opinions being rejected				0.414	
Factor 5: Workplace resources and stability					
OJFQ 35 Worries about losing my job make me less satisfied with my current position					0.794
OJFQ 28 I feel that congested offices with a lack of privacy are not conducive to achieving tasks comfortably					0.576
OJFQ 36 My ability to tolerate crises greatly influences my stress levels at work					0.494
OJFQ 34 Lacking of expert in my field causing me stress					0.456

The internal consistency of the OJFQ was acceptable, with a Cronbach’s alpha of 0.774 for the total scale. Subscale reliability coefficients ranged from 0.672 to higher values, indicating acceptable to good reliability for a newly developed multidimensional instrument.

Among the five factors in the extracted findings within the EFA, “management and role clarity” factor represents the highest average score with 48.6 ± 9.8, followed by “workplace bureaucracy and social challenges” with average score of 36.1 ± 6.8. The internal consistency of the OJFQ as a whole measured with Cronbach’s alpha is 0.774 where all subscales presented acceptable to good reliability and the lowest score was 0.672 for “workplace resources and stability” which is still considered as acceptable at the scale development phase (Table 7). The observed inter-factor correlations further support the conceptualization of job frustration as a multidimensional construct with interconnected domains rather than independent components. It should be noted that all reported reliability estimates refer to the final 37-item version of the OJFQ following item reduction and factor refinement.

**Table 7:** Reliability of the OJFQ (*N* = 139)

Components	No. of Items	Mean	SD	Cronbach’s Alpha	Correlation between 5 factors and total score^a^	Composite Reliability (CR)	Interpretation
Total score of the questionnaire	37	179.05	22.096	0.774			
Factor 1: Management and role clarity	10	48.64	9.781	0.820	.825**	0.8337	Good
Factor 2: Emotional regulation and coping responses	8	20.95	4.688	0.821	.183*	0.8333	Good
Factor 3: Career development and engagement	8	22.21	7.161	0.811	.437**	0.8468	Excellent
Factor 4: Workplace bureaucracy and social challenges	7	36.10	6.793	0.785	.635**	0.7630	Acceptable
Factor 5: Workplace resources and stability	4	16.82	3.224	0.672	.347**	0.6755	Moderate

^a^Spearman’s rank correlation coefficient

**p < 0.01

#### Confirmatory factor analysis and construct and convergent validity

**Fig. 3:**
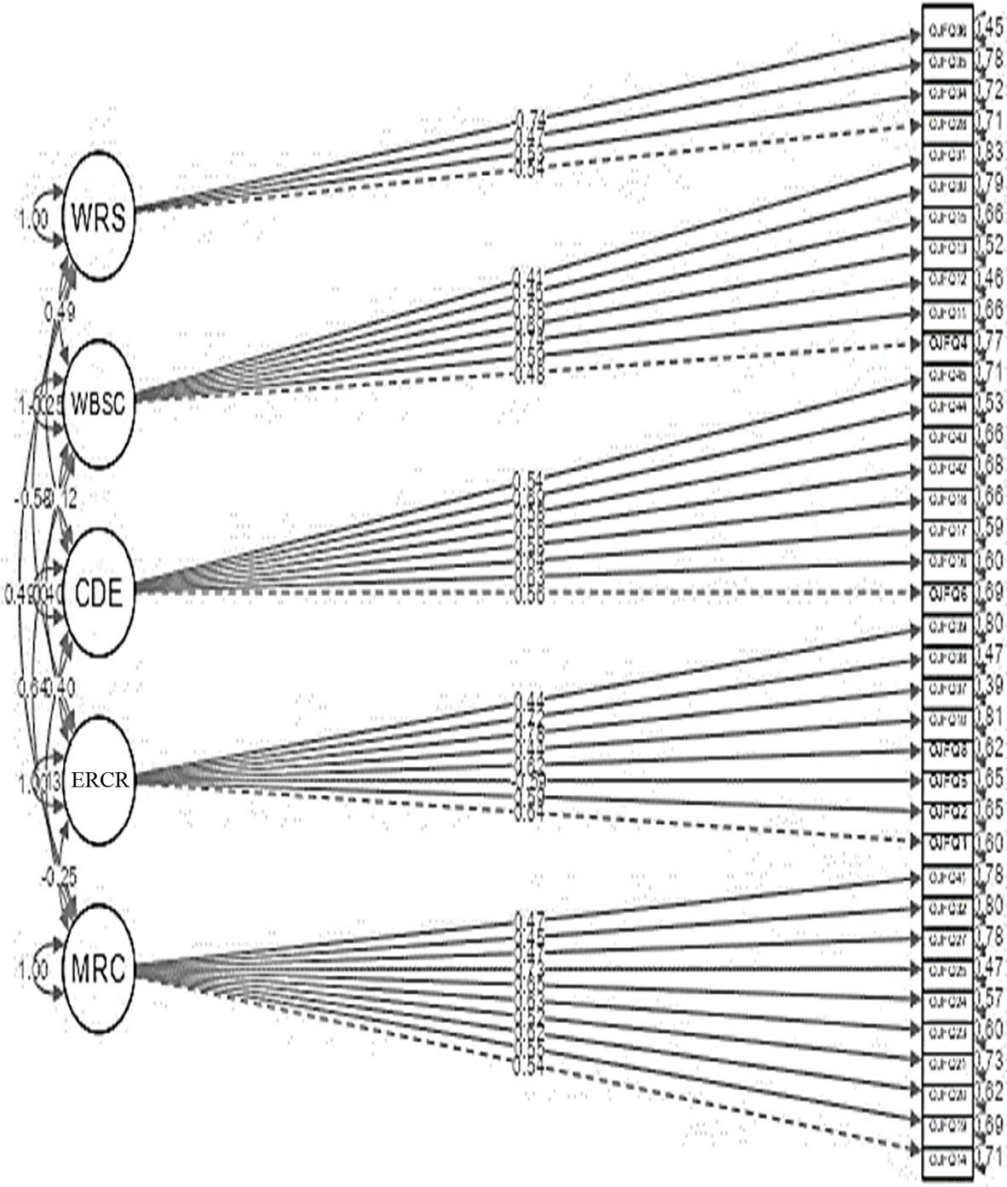
Confirmatory factor analysis model of the OJFQ

CFA was conducted using structural equation modelling to evaluate the five-factor structure identified in the exploratory factor analysis. The model was assessed using the unweighted least squares estimate. Several model fits indicated suboptimal model fit (Table 8). The findings provided preliminary support for five-factor model as preliminary due to weak goodness of fit of the newly developed scale OJFQ (Table 8 and Fig. 3).

**Table 8:** Construct validity of the OJFQ

Five-Factor Model	X2/df	GFI	AGFI	NNFI	NFI	CFI	IFI	RFI	PNFI	PGFI
Scale	1199/619 = 1.9	0.689	0.647	0.651	0.511	0.675	0.684	0.474	0.475	0.607

*X2* Chi-square, *df* Degrees of freedom, *GFI* Goodness of fit index, *AGFI* Adjusted goodness of fit index, *NNFI* Non-normed fit index, *NFI* Normed fit index, *CFI* Comparative fit index, *IFI* Incremental fit index, *RFI* Relative fit index, *PNFI* Parsimony normed fit index, *PGFI* Parsimony goodness of fit index

Figure 3 is a model diagram of CFA representing the structure of the final validation of OJFQ illustrating the latent factor structure and their corresponding items with loadings. In the diagram, all factor loadings demonstrating acceptable association between items and their respective construct which are above the recommended threshold of 0.40. The figure illustrates the correlation between constructs where the standardized loading ranged from 0.47 to 0.83 suggesting acceptable item-factor relationship.

**Fig. 4:**
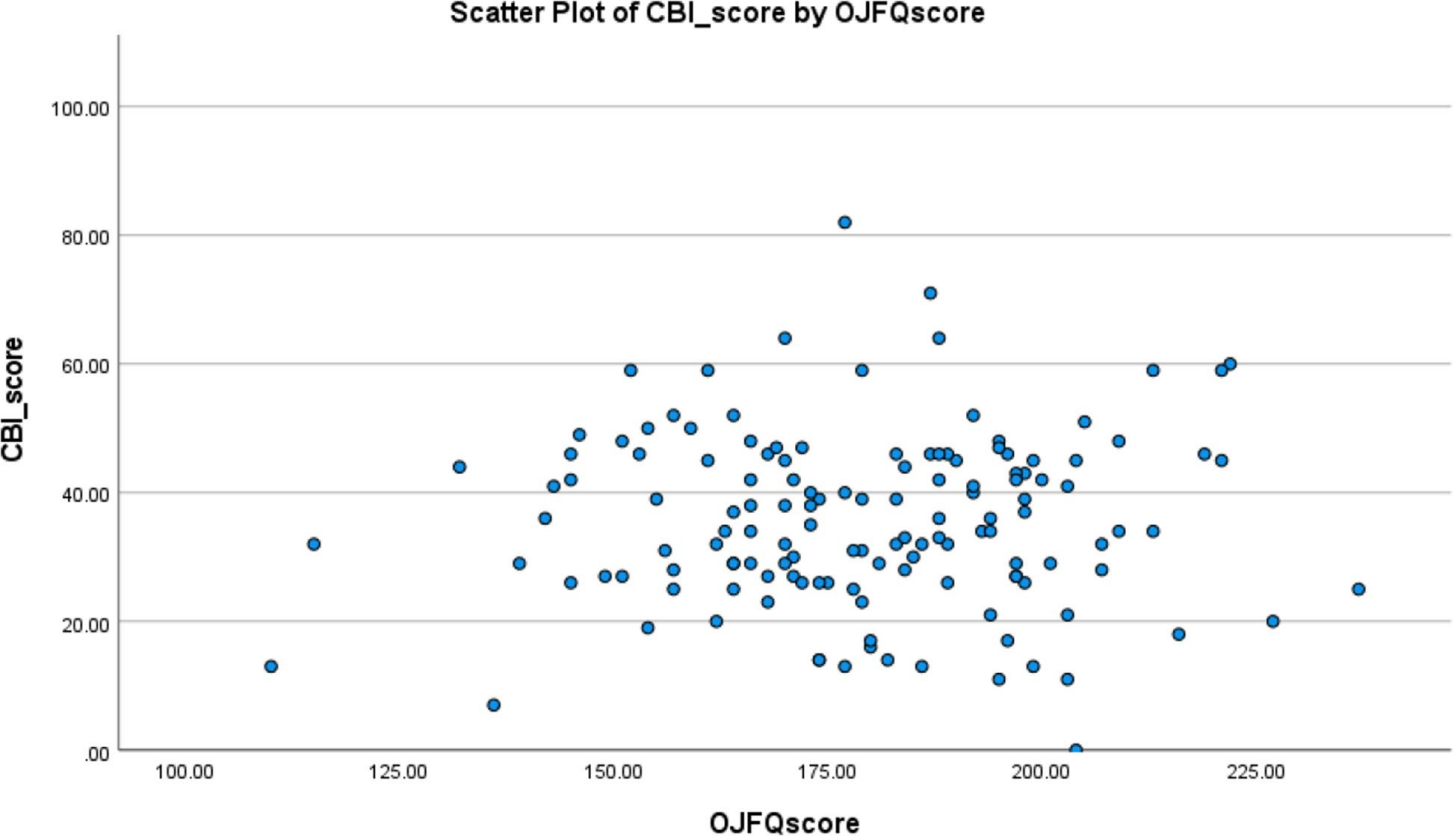
Scatter plot of OJFQ and CBI scores

Figure 4 shows the convergent validity through scatter plot of OJFQ and the CBI indicating no visible linear trend supporting non-significant correlation (*r* = 0.032, *p* = 0.709). The Pearson’s correlation analysis suggested that the OJFQ may assess dimensions distinct from those measure by the CBI.

The scores of PGFI (0.607) and PNFI (0.475) were within acceptable range indicating a reasonable model complexity balance. However, the remaining fit indices scores fell below the acceptable range which is 0.90. Since the Chi-square ratio indicates satisfactory fit, the refinement of the model structure may be suggested to improve model representation of the observed data. The CFA was conducted on the same sample used for EFA, and therefore these findings provide preliminary support for the factor structure. Overall, the CFA results provide initial support for the multidimensional structure of the OJFQ while highlighting the need for further model refinement and validation.

#### Correlation with Copenhagen burnout inventory (CBI)

Pearson’s correlation analysis was conducted to examine the relationship between OJFQ and CBI scores. The results indicated that the OJFQ total score was not significantly correlated with the CBI total score (*r* = 0.032, *p* = 0.709). This suggests that the OJFQ may capture a construct that is distinct from burnout as measured by the CBI.

These findings reflect the complex and context-specific nature of job frustration, especially in culturally oriented healthcare environments, where newly developed culturally grounded instruments often require iterative psychometric refinement.

## Discussion

The findings of this study support the conceptualization of job frustration as a distinct and multidimensional construct, rather than a substitution for burnout or general occupational stress. The identified domains reflect contextually grounded experiences of workplace barriers, organizational challenges, and emotional responses, consistent with the qualitative phase of this study. By distinguishing job frustration from related constructs, the OJFQ provides a more theoretically proximal and potentially more sensitive measure of early occupational psychological strain, permitting earlier detection of workplace difficulties before they evolve into more chronic conditions such as burnout. This reinforces the value of focusing on job frustration as an independent construct within occupational health research, particularly in complex healthcare settings.

The scale validation process initially generated a broad pool of potential items derived from participant response during the exploratory phase (qualitative phase) which underwent expert review by the research team and subject-matter specialists. This was followed by psychometric evaluation using EFA and CFA. In addition, item retention decisions were based not only on statistical indicators, but also on conceptual coherence, theoretical relevance, and cultural applicability within the Omani healthcare context. Therefore, certain items with marginal psychometric performance were provisionally retained due to their theoretical relevance, contributing to construct coverage, and contextual significance within the Omani healthcare setting. As a result, a 37-item Omani Job Frustration Questionnaire (OJFQ) was developed highlighting five factors which are: (1) “management and role clarity”, (2) “emotional regulation and professional coping strategies”, (3) “career development and engagement”, (4) “workplace bureaucracy and social challenges”, and (5) “workplace resources and stability”. The identified factor structure appears conceptually consistent with the qualitative themes generated during the exploratory phase of the study, particularly themes related to organizational management, emotional coping, workplace bureaucracy, and professional development.

Effective organizational management relies on the use of measurable indicators to identify and address workplace challenges [33]. Therefore, in order to enhance the work commitment and employee well-being, regular assessment of job frustration may facilitate early identification of occupational strain and support employee well-being. Additionally, the scale development and validation process indicated that the HCWs appeared capable of tolerating occupational workload demands, whereas psychological, social, and organizational factors exerted greater influence on overall performance. Therefore, the focus on the underlying contributing factors can assist the managers to define the approach. Similarly, limited variation across specialities was observed in this sample, though further investigation is needed.

The identified factors supported multidimensional, context-specific nature of job frustration and demonstrated alignment with empirical findings from Oman and other GCC countries [4, 15, 17, 34–41] and other GCC research findings [2, 6, 16, 18, 42–45]. The factor supported recommendations from evidences highlighting effects of demanding nature of work, limited career development or promotion opportunities, management support, reward system, work-life balance, inter-personal relationship, foreign HCWs’ perceptions of occupational insecurity associated with workforce localization national policy and available coping measures. Some items within the finding of the study related to emotional regulation, organizational fairness, and religious coping are elements collectively representing a unified coping domain reflecting how nurses regulate emotional stressors within their cultural and organizational context. Therefore, unlike standardized available scales, the OJFQ is developed from locally derived narratives and culturally contextualized workplace experiences tailored, explores localized policy impact, context-specific, workplace structure relevance and highlights cultural related dynamics in the Omani context.

The findings supported the statistical adequacy of the data for factor analysis and provided initial evidence of the scale’s psychometric properties. This had indicated that the data were suitable for factor analysis. Additionally, the Cronbach’s alpha of the final scale and subscales, along with composite reliability values, demonstrated good to moderate internal consistency, with several subscales demonstrating marginal yet acceptable reliability for an exploratory scale-development study. While some items demonstrated lower factor loadings, the overall multidimensional structure remained theoretically coherent and aligned with the underlying constructs. The instrument demonstrated preliminary evidence of construct validity, although model fit indices suggested that further refinement of the factor structure may improve overall model adequacy. Future studies should further evaluate discriminant, and predictive validity using external psychological and occupational measures.

The findings indicate acceptable internal consistency and initial psychometric support. The score of the cumulative variance of the scale sub-divisions indicates good representation of the factors within the scale. The scale evaluation indicated that the reliable average score and good to acceptable internal reliability shown in constructs representing social challenges, management and workplace bureaucracy. This may reflect culturally relevant dimensions of occupational experience within the Omani healthcare context. Therefore, the OJFQ shows promise as a contextually relevant assessment tool, warranting further validation before broader application. The absence of a statistically significant correlation with the Copenhagen Burnout Inventory (CBI) (*r* = 0.032, *p* = 0.709) provides preliminary support for the discriminant distinction between job frustration and burnout.

This scale would contribute to the contextualized assessment approach being the one of the first culturally tailored instruments with emerging psychometric support to measure the job frustration particularly in healthcare environments where occupational strain may be experienced and expressed through understated frustration-related responses before progression to overt burnout manifestations. It will further improve mental health support through implementing the assessment scale. This study is considered as a foundation contribution for regional and other relevant researches as well as would be considered as cross-cultural research contribution in non-western regions.

It is important to note that the current study was conducted within a mental health care setting and primarily involved nursing professionals. Therefore, the findings may reflect context-specific experiences of job frustration that are particularly relevant to this group. While the identified factors may have broader relevance, the generalizability of the OJFQ to other healthcare disciplines and settings remains to be established.

Despite the significant insights generated by this study, there are notable limitations acknowledged. Firstly, the study sample was context-specific which was conducted in mental health setting among nursing professions only. Therefore, the sample was limited to mental health nursing professionals, which may restrict the generalizability of the findings to other healthcare disciplines, specialties, and clinical settings. A broader group of participants may be considered to improve applicability through cross-validation across different healthcare settings and professional groups. Secondly, a key methodological limitation of this study is that both exploratory factor analysis (EFA) and confirmatory factor analysis (CFA) were conducted using the same sample. While this approach is sometimes used in early-stage scale development, it limits the strength of confirmatory validation and increases the risk of overfitting the model to the sample data. Ideally, CFA should be conducted on an independent sample to provide stronger evidence of construct validity. Therefore, the current findings should be interpreted as preliminary, and future studies are needed to replicate and validate the factor structure in larger and independent samples.

Thirdly, response bias may have been introduced due to the frequent encouragement from the researchers and data collectors to the participants to respond resulting in potential skewness of the findings. Fourthly, cultural influences may have affected the participant responses especially within the Arab cultural background where challenges are mostly underreported and may lead to social desirability bias. It is recommended to consider other data collection approaches such as confidential individual interviews. Finally, recall bias as well as subjective interpretation may be imposed due to the reliance on self-reporting in groups requiring triangulation of the findings with for instance observation.

## Conclusion

The healthcare researches on employee job frustration in Oman are scarce and old. Most of these researches depend on international scales designed in western culture. Accurate assessment of job frustration is necessary to support effective organizational management and early identification of job frustration. Therefore, the need to develop a culturally relevant scale is set and put forward to help policy makers and manager to consider regular assessment of the mental state of employee towards their job. Although the psychometric findings do not yet support a definitive model, it is considered an acceptably reliable instrument with preliminary evidence of validity, requiring further refinement in future researches. In addition, as both exploratory and confirmatory factor analyses were conducted on the same sample, the validation findings should be interpreted as preliminary and warrant confirmation using independent samples. Therefore, the newly developed instrument should be applied cautiously beyond comparable clinical settings pending further psychometric validation.

## Supplementary Information

Supplementary Material 1.

Supplementary Material 2.

## Data Availability

The datasets used and/or analysed during the current study are available from the corresponding author on reasonable request.

## Abbreviations

OJFQ: Omani Job Frustration Questionnaire
UNIMAS: Universiti Malaysia Sarawak
CBI: Copenhagen Burnout Inventory
MBI: Maslach Burnout Inventory
HCWs: Health Care Workers
FGDs: Focused Group Discussions
EFA: Exploratory Factor Analysis
CFA: Confirmatory Factor Analysis
CVI: Content Validity Index
CR: Composite Reliability
SD: Standard Deviation
PGFI: Parsimony goodness of fit index
PNFI: Parsimony normed of fit index
