# Supplementary material for: Measuring job frustration in Omani healthcare workers: development and psychometric validation of the OJFQ
Source: BMC Psychol. 2026 May 19;14:1029. doi: 10.1186/s40359-026-04762-5 (PMC13352741; doi:10.1186/s40359-026-04762-5)

**Table 1. Incorporation of MARS Model with Six-Step Process of Braun and Clarke (2022) of Thematic Analysis**

| **Phase** | **Direction** | **Actual Action** |
| --- | --- | --- |
| **Phase 1** | Getting to Know the Data | Reading through the qualitative data obtained from FGDs to familiarize with it. Clear out instances in which participants bring up elements of the MARS model, such as:   - situational factors (e.g., poor work environment), - role perception (e.g., unclear job expectations), - ability (e.g., feeling underqualified), - or motivation (e.g., lack of rewards) |
| **Phase 2** | Making the first codes | As to begin to code the data, the codes are created that reflect the MARS factors. For example, codes could include terms like "Undervalued reward system," "role clarity," "insufficient resources," or "negative work conditions." |
| **Phase 3** | Searching for themes | The codes would be grouped into more general categories surfacing the MARS framework. A collection of codes pertaining to "lack of motivation" can, for example, create a topic regarding employee disengagement. Similarly, a theme about inadequate training and development may be formed by codes pertaining to capacity (e.g., role perception and clarity). |
| **Phase 4** | Examining the themes | The themes would be examined and improved to make sure they appropriately depict the facts. They can be combined or reinterpreted themes of motivation (like irritation over unclear roles) if they appear to overlap with role perception themes (like confusion about job tasks) to ensure that they clearly relate to the MARS model. |
| **Phase 5** | Themes definition and naming | Each theme would explicitly be outlined after being decided on them. "Frustration Due to Role Ambiguity," for instance, may be a theme that closely relates to role perception according to the MARS model. The theoretical foundation of each theme is ensured by connecting them to MARS. |
| **Phase 6** | Putting together the report | Writing the analysis would include making links between the MARS model and the themes that were found. It would describe how issues like low motivation or a lack of skills lead to job dissatisfaction and provide data examples to show how each MARS factor appears in workers' experiences. |

**Table 2. List of items for reverse scoring**

| **Item Number** | **Statement** |
| --- | --- |
| N 1 | My emotional well-being significantly improves when a manager demonstrates empathy. |
| N 2 | My understanding of the stressors enhances my affective state |
| N 3 | I can effectively reduce stress by openly discussing my concerns with my managers |
| N 6 | My performance improves with the presence of supervisors. |
| N 8 | My level of commitment to task is enhanced when I involve in decision-making |
| N 10 | I feel less frustrated when the workload is distributed fairly in my workplace. |
| N 16 | My current working environment is safe for me to practice new skills |
| N 17 | I am having a supportive relationship with my leader that reduce my job frustration and improves my performance |
| N 18 | I am getting positive reinforcement and encouragement at work like everyone else |
| N 36 | My ability to tolerate crises greatly influences my stress levels at work |
| N 37 | My resilience at work improves when I can effectively understand and manage emotions |
| N 38 | My ability to recognize emotions in myself and others improves my coping skills |
| N 39 | My faith or religious beliefs provide me with strength to cope with work-related stress |
| N 40 | Having strong support from my family helps to reduce my job dissatisfaction |
| N 42 | I receive constructive feedback that help improving my skills, performance and learning that lead to less job frustration |
| N 43 | I am actively participating in own learning process to reduce my job frustration |
| N 44 | My training materials are directly applicable to my daily tasks that reduce my job frustration |
| N 45 | I feel that staying updated with the latest trends in healthcare helps me manage my job frustration |

**Table 3. Demographic characteristics of the participants (N=139)**

| Item | N (%) | Mean |  | SD |
| --- | --- | --- | --- | --- |
| **Age (years)** | - | 33.18 | + | 6.207 |
| **Work experience (years)** | - | 9.72 | + | 5.803 |
| **Gender** |  |  |  |  |
| Male | 67 (47.5%) |  |  |  |
| Female | 73 (51.8%) |  |  |  |
| **Field of practice** |  |  |  |  |
| Clinical | 61 (43.3%) |  |  |  |
| Leadership | 77 (54.6%) |  |  |  |

**Table 4. Item analysis of OJFQ (N= 139)**

| **Items** | **Mean** | **SD** | **Skewness** | | **Kurtosis** | | **t** | **95% CI** | | **Correlation with total score** |
| --- | --- | --- | --- | --- | --- | --- | --- | --- | --- | --- |
|  |  |  | **Statistic** | **Std. Error** | **Statistic** | **Std. Error** |  | **Lower** | **Upper** |  |
| **Total Score of Scale** | **179.0504** | **22.09608** | **-.222-** | **.206** | **.322** | **.408** | **95.536** | **175.3446** | **182.7562** | **1** |
| **Q1** | **2.17** | **1.258** | **1.387** | **.205** | **2.114** | **.407** | **20.428** | **1.96** | **2.38** | **.168*** |
| **Q2** | **2.09** | **1.042** | **1.297** | **.205** | **1.158** | **.407** | **23.680** | **1.91** | **2.26** | **.238**** |
| **Q3** | **2.71** | **1.407** | **1.055** | **.205** | **.810** | **.407** | **22.773** | **2.47** | **2.94** | **.280**** |
| **Q4** | **5.33** | **1.476** | **-1.020-** | **.205** | **.489** | **.407** | **24.711** | **5.08** | **5.58** | **.411**** |
| **Q5** | **5.70** | **1.216** | **-1.525-** | **.205** | **2.808** | **.407** | **55.481** | **5.50** | **5.90** | **.107** |
| **Q6** | **3.16** | **1.576** | **.966** | **.205** | **.018** | **.407** | **23.757** | **2.90** | **3.43** | **.198*** |
| **Q7** | **4.83** | **1.799** | **-.942-** | **.205** | **-.241-** | **.407** | **31.757** | **4.53** | **5.13** | **.136** |
| **Q8** | **2.06** | **1.051** | **1.431** | **.205** | **3.084** | **.407** | **23.159** | **1.88** | **2.23** | **.110** |
| **Q9** | **5.62** | **1.354** | **-1.293-** | **.205** | **1.299** | **.407** | **49.114** | **5.40** | **5.85** | **.182*** |
| **Q10** | **2.46** | **1.305** | **1.110** | **.205** | **.645** | **.407** | **22.277** | **2.24** | **2.68** | **-.036-** |
| **Q11** | **5.13** | **1.559** | **-.980-** | **.205** | **.162** | **.407** | **38.932** | **4.87** | **5.39** | **.322**** |
| **Q12** | **5.36** | **1.425** | **-1.075-** | **.205** | **.841** | **.407** | **44.528** | **5.13** | **5.60** | **.450**** |
| **Q13** | **4.86** | **1.532** | **-.669-** | **.205** | **-.263-** | **.407** | **37.558** | **4.61** | **5.12** | **.583**** |
| **Q14** | **4.92** | **1.532** | **-.622-** | **.205** | **-.279-** | **.407** | **38.016** | **4.67** | **5.18** | **.513**** |
| **Q15** | **5.24** | **1.478** | **-.984-** | **.205** | **.355** | **.407** | **41.965** | **5.00** | **5.49** | **.267**** |
| **Q16** | **3.02** | **1.620** | **.963** | **.205** | **-.041-** | **.407** | **22.064** | **2.75** | **3.29** | **.490**** |
| **Q17** | **2.38** | **1.135** | **1.160** | **.205** | **1.135** | **.407** | **24.805** | **2.19** | **2.57** | **.502**** |
| **Q18** | **2.86** | **1.447** | **.773** | **.205** | **-.296-** | **.407** | **23.359** | **2.62** | **3.10** | **.656**** |
| **Q19** | **4.44** | **1.855** | **-.391-** | **.205** | **-1.141-** | **.407** | **28.290** | **4.13** | **4.75** | **.649**** |
| **Q20** | **4.64** | **1.734** | **-.450-** | **.205** | **-.853-** | **.407** | **31.673** | **4.35** | **4.93** | **.495**** |
| **Q21** | **4.58** | **1.650** | **-.398-** | **.205** | **-.869-** | **.407** | **32.741** | **4.31** | **4.86** | **.257**** |
| **Q22** | **4.28** | **1.957** | **-.252-** | **.205** | **-1.260-** | **.407** | **25.874** | **3.95** | **4.61** | **.519**** |
| **Q23** | **5.10** | **1.416** | **-.581-** | **.205** | **-.206** | **.407** | **42.624** | **4.86** | **5.34** | **.460**** |
| **Q24** | **5.06** | **1.425** | **-.568-** | **.205** | **-.187-** | **.407** | **42.038** | **4.83** | **5.30** | **.364**** |
| **Q25** | **4.89** | **1.455** | **-.638-** | **.205** | **-.218-** | **.407** | **39.737** | **4.64** | **5.13** | **.333**** |
| **Q26** | **4.79** | **1.492** | **-.482-** | **.205** | **-.476** | **.407** | **37.944** | **4.54** | **5.04** | **.392**** |
| **Q27** | **4.26** | **1.922** | **-.229-** | **.205** | **-1.306-** | **.407** | **26.258** | **3.94** | **4.59** | **.320**** |
| **Q28** | **5.05** | **1.538** | **-.832-** | **.205** | **-.004-** | **.407** | **38.859** | **4.79** | **5.31** | **.225**** |
| **Q29** | **4.71** | **1.557** | **-.486-** | **.205** | **-.535-** | **.407** | **35.770** | **4.45** | **4.97** | **-.135-** |
| **Q30** | **4.91** | **1.586** | **-.679-** | **.205** | **-.443-** | **.407** | **36.601** | **4.64** | **5.17** | **.310**** |
| **Q31** | **5.26** | **1.506** | **-.858-** | **.205** | **-.131-** | **.407** | **41.362** | **5.01** | **5.52** | **.295**** |
| **Q32** | **5.67** | **1.375** | **-1.122-** | **.205** | **.917** | **.407** | **48.796** | **5.44** | **5.90** | **.268**** |
| **Q33** | **5.48** | **1.486** | **-1.132-** | **.205** | **.515** | **.407** | **43.623** | **5.23** | **5.73** | **.384**** |
| **Q34** | **4.89** | **1.539** | **-.718** | **.205** | **-.468-** | **.407** | **37.608** | **4.64** | **5.15** | **.299**** |
| **Q35** | **4.23** | **1.715** | **-.223-** | **.205** | **-1.005-** | **.407** | **29.179** | **3.94** | **4.52** | **.266**** |
| **Q36** | **2.65** | **1.175** | **1.172** | **.205** | **1.234** | **.407** | **26.689** | **2.45** | **2.85** | **.582**** |
| **Q37** | **2.21** | **.973** | **.983** | **.205** | **1.119** | **.407** | **26.929** | **2.05** | **2.38** | **.583**** |
| **Q38** | **2.31** | **1.150** | **1.361** | **.205** | **2.070** | **.407** | **23.740** | **2.11** | **2.50** | **.515**** |
| **Q39** | **1.96** | **1.024** | **1.473** | **.205** | **2.602** | **.407** | **22.615** | **1.79** | **2.13** | **.636**** |
| **Q40** | **1.96** | **1.045** | **1.507** | **.205** | **2.599** | **.407** | **22.164** | **1.78** | **2.13** | **.027** |
| **Q41** | **5.04** | **1.416** | **-.680-** | **.205** | **-.104-** | **.407** | **42.070** | **4.80** | **5.27** | **.119** |
| **Q42** | **2.94** | **1.358** | **.974** | **.205** | **.617** | **.407** | **25.575** | **2.71** | **3.16** | **.034** |
| **Q43** | **2.70** | **1.262** | **1.173** | **.205** | **1.295** | **.407** | **25.313** | **2.49** | **2.91** | **.087** |
| **Q44** | **2.81** | **1.267** | **.787** | **.205** | **-.034-** | **.407** | **26.280** | **2.60** | **3.03** | **.288**** |
| **Q45** | **2.34** | **1.174** | **1.197** | **.205** | **1.662** | **.407** | **23.615** | **2.15** | **2.54** | **.285**** |

****. Correlation is significant at the 0.01 level (2-tailed).**

***. Correlation is significant at the 0.05 level (2-tailed).**

**Table 5. Item-Level Content Validity Index (I-CVI) and Modified Kappa for OJFQ (merged phase 1 and phase 2 expert panel review)**

| **Item** | **N** | **A** | **CVI** | **Pc** | **Modified Kappa (K)** | **Interpretation of modified kappa (K)** |
| --- | --- | --- | --- | --- | --- | --- |
| **Factor 1: Management and role clarity** |  |  |  |  |  |  |
| 1 I get frustrated when colleagues mix up their job duties with their personal relationships with leaders. | 15 | 9 | 0.2 | 0.00030 | 0.198 | Poor |
| 2 I feel lack of confidence due to poorly defined job descriptions and task allocations among different members of the healthcare multidisciplinary team | 15 | 14 | 0.87 | 0.00046 | 0.870 | Excellent |
| 3 there is extreme interference from top management in leaders’ tasks. | 15 | 12 | 0.6 | 0.0139 | 0.594 | Good |
| 4 there is a waste of effort in succession planning since the selection of new healthcare leaders comes solely from top authorities | 15 | 11 | 0.47 | 0.04166 | 0.447 | Fair |
| 5 My concerns are acknowledged by the top management, but no substantial actions are taken to address them. | 15 | 13 | 0.73 | 0.04166 | 0.718 | Good |
| 6 it is hard to change basic policies in the hospital to adapt to the dynamicity of technological advances | 16 | 12 | 0.5 | 0.02777 | 0.486 | Fair |
| 7 My current working environment is safe for me to practice new skills (suggested) |  |  |  |  |  |  |
| 8. I am having a supportive relationship with my leader that reduce my job frustration and improves my performance (suggested) |  |  |  |  |  |  |
| 9. I receive constructive feedback that help improving my skills, performance and learning that lead to less job frustration (suggested) |  |  |  |  |  |  |
| 10. I am actively participating in own learning process to reduce my job frustration (suggested) |  |  |  |  |  |  |
| 11. My training materials are directly applicable to my daily tasks that reduce my job frustration (suggested) |  |  |  |  |  |  |
| 12. I feel that staying updated with the latest trends in healthcare helps me manage my job frustration (suggested) |  |  |  |  |  |  |
| 13 My emotional well-being significantly improves when a manager demonstrates empathy. | 16 | 14 | 0.75 | 0.00183 | 0.750 | Excellent |
| 14 level of commitment to task is enhanced when I involve in decision-making | 15 | 15 | 1.00 | 0.00003 | 1.00 | Excellent |
| 15. My resilience at work improves when I can effectively understand and manage emotions | 16 | 15 | 0.88 | 0.00024 | 0.880 | Excellent |
| 16. I feel less frustrated when the workload is distributed fairly in my workplace. | 15 | 15 | 1.00 | 0.00003 | 1.00 | Excellent |
| 17. My frustration caused by unclear/ lack of policies and procedures | 15 | 13 | 0.73 | 0.04166 | 0.718 | Good |
| 18. I feel overwhelmed doing additional administrative responsibilities under unhelpful management policies. | 15 | 13 | 0.73 | 0.04166 | 0.718 | Good |
| 19. I am frustrated by the demanding administration works | 15 | 13 | 0.73 | 0.04166 | 0.718 | Good |
| 20. I am often blamed when a clinical-related adverse incident occurs | 15 | 13 | 0.73 | 0.04166 | 0.718 | Good |
| 21. If I accept extra work responsibilities, I will be blamed for the extra work not being done as required | 16 | 13 | 0.63 | 0.00854 | 0.627 | Good |
| 22. I have fear of blame while making decision or writing incident reports | 15 | 13 | 0.73 | 0.04166 | 0.718 | Good |
| 23. There is gender stereotype in healthcare related fields that stressed me up | 16 | 15 | 0.88 | 0.00024 | 0.880 | Excellent |
| 24. I need to put more efforts to balance between professional and personal life | 16 | 13 | 0.63 | 0.00854 | 0.627 | Good |
| 25. Working on shift duties is affecting my social life | 16 | 14 | 0.75 | 0.00183 | 0.750 | Excellent |
| 26. I can effectively reduce stress by openly discussing my concerns with my managers | 16 | 15 | 0.88 | 0.00024 | 0.880 | Excellent |
| 27. My ability to tolerate crises greatly influences my stress levels at work | 14 | 13 | 0.86 | 0.00085 | 0.860 | Excellent |
| 28. My ability to recognize emotions in myself and others improves my coping skills | 15 | 12 | 0.6 | 0.0139 | 0.594 | Fair |
| 29. My faith or religious beliefs provide me with strength to cope with work-related stress (suggested) |  |  |  |  |  |  |
| 30. Having strong support from my family helps to reduce my job dissatisfaction (Suggested) |  |  |  |  |  |  |
| 31. I feel demotivated when coming to work with all requests or opinions being rejected | 16 | 16 | 1.00 | 0.000015 | 1.00 | Excellent |
| 32. I as a healthcare worker prefer to remain silent at work | 14 | 12 | 0.71 | 0.00555 | 0.708 | Good |
| 33. I feel that congested offices with a lack of privacy are not conducive to achieving tasks comfortably. | 15 | 14 | 0.87 | 0.00046 | 0.870 | Excellent |
| 34. I encounter physical risks while working in the mental health field. | 16 | 14 | 0.75 | 0.00183 | 0.750 | Excellent |
| 35. I am at risk of developing mental health challenges as a result of my work in this field. | 16 | 14 | 0.75 | 0.00183 | 0.750 | Excellent |
| 36. Worries about losing my job make me less satisfied with my current position | 17 | 13 | 0.53 | 0.018157 | 0.521 | Fair |
| 37. I am stressed when there is lack of teamwork | 16 | 13 | 0.63 | 0.00854 | 0.627 | Good |

| **Note**:  ◦ The formula for modified kappa statistic (κ) = (CVI– pc)/ (1– pc), where pc represents probability of a chance occurrence  ◦ P_c_ is the probability of chance of occurrence. The formula for pc is: N! / [A! *(N-A)!] *0.5^N^ where N = the number of judges, A = the number agreeing on good relevance and recommended  ◦ Evaluation criteria for modified kappa (κ): κ = poor (< 0.40), κ = fair (0.40–0.59), κ = good (0.60–0.74) and κ = excellent (> 0.74)  ◦ CVI should be 0.88 and above to establish validity with a *p* < 0.05, CVI <0.49 has to be rejected |  |
| --- | --- |

**Table 6. Rotated factors for principle component analysis of OJFQ**

| **Items** | **Factor Loading** | | | | |
| --- | --- | --- | --- | --- | --- |
|  | **I** | **II** | **III** | **IV** | **V** |
| **Factor 1: Management and role clarity** |  |  |  |  |  |
| OJFQ **24** There is a waste of effort in succession planning since the selection of new healthcare leaders comes solely from top authorities. | 0.745 |  |  |  |  |
| OJFQ **25** My concerns are acknowledged by the top management, but no substantial actions are taken to address them. | 0.713 |  |  |  |  |
| OJFQ **23** There is extreme interference from top management in leaders’ tasks. | 0.702 |  |  |  |  |
| OJFQ **41** It is hard to change basic policies in the hospital to adapt to the dynamicity of technological advances | 0.578 |  |  |  |  |
| OJFQ **19** I am often blamed when a clinical-related adverse incident occurs | 0.570 |  |  |  |  |
| OJFQ **32** Working on shift duties is affecting my social life | 0.521 |  |  |  |  |
| OJFQ **21** Shift supervisors are reluctant to make clinical decisions without consulting their immediate manager | 0.493 |  |  |  |  |
| OJFQ**14** There is unclear delineation of roles among different members of the healthcare multidisciplinary team | 0.477 |  |  |  |  |
| OJFQ**20** If I accept extra work responsibilities, I will be blamed for the extra work not being done as required | 0.476 |  |  |  |  |
| OJFQ**27** I as a healthcare worker prefer to remain silent at work | 0.474 |  |  |  |  |
| **Factor 2: Emotional regulation and coping responses** |  |  |  |  |  |
| OJFQ **8** My level of commitment to task is enhanced when I involve in decision-making |  | **0.751** |  |  |  |
| OJFQ **37** My resilience at work improves when I can effectively understand and manage emotions |  | **0.712** |  |  |  |
| OJFQ1 My emotional well-being significantly improves when a manager demonstrates empathy. |  | **0.684** |  |  |  |
| OJFQ **5** I am stressed when there is lack of teamwork |  | **0.679** |  |  |  |
| OJFQ **38** My ability to recognize emotions in myself and others improves my coping skills |  | **0.618** |  |  |  |
| OJFQ **2** My understanding of the stressors enhances my affective state |  | **0.577** |  |  |  |
| OJFQ **10** I feel less frustrated when the workload is distributed fairly in my workplace. |  | **0.510** |  |  |  |
| OJFQ **39** My faith or religious beliefs provide me with strength to cope with work-related stress |  | **0.400** |  |  |  |
| **Factor 3: Career development and engagement** |  |  |  |  |  |
| OJFQ **44** My training materials are directly applicable to my daily tasks that reduce my job frustration |  |  | **0.735** |  |  |
| OJFQ **43** I am actively participating in own learning process to reduce my job frustration |  |  | **0.730** |  |  |
| OJFQ **42** I receive constructive feedback that help improving my skills, performance and learning that lead to less job frustration |  |  | **0.705** |  |  |
| OJFQ **16** My current working environment is safe for me to practice new skills |  |  | **0.674** |  |  |
| OJFQ **45** I feel that staying updated with the latest trends in healthcare helps me manage my job frustration |  |  | **0.631** |  |  |
| OJFQ **17** I am having a supportive relationship with my leader that reduce my job frustration and improves my performance |  |  | **0.588** |  |  |
| OJFQ **18** I am getting positive reinforcement and encouragement at work like everyone else |  |  | **0.530** |  |  |
| OJFQ **6** My performance improves with the presence of supervisors. |  |  | **0.503** |  |  |
| **Factor 4: Workplace bureaucracy and social challenges** |  |  |  |  |  |
| OJFQ **12** I feel overwhelmed doing additional administrative responsibilities under unhelpful management policies. |  |  |  | **0.674** |  |
| OJFQ **30** There is gender stereotype in healthcare related fields that stressed me up |  |  |  | **0.639** |  |
| OJFQ **11** My frustration caused by unclear/ lack of policies and procedures |  |  |  | **0.598** |  |
| OJFQ **13** I am frustrated by the demanding administration works |  |  |  | **0.547** |  |
| OJFQ **31** I need to put more efforts to balance between professional and personal life |  |  |  | **0.500** |  |
| OJFQ **15** I feel lack of confidence due to poorly defined job descriptions and task allocations among different members of the healthcare multidisciplinary team |  |  |  | **0.495** |  |
| OJFQ **4** I feel demotivated when coming to work with all requests or opinions being rejected |  |  |  | **0.414** |  |
| **Factor 5: Workplace resources and stability** |  |  |  |  |  |
| OJFQ **35** Worries about losing my job make me less satisfied with my current position |  |  |  |  | **0.794** |
| OJFQ **28** I feel that congested offices with a lack of privacy are not conducive to achieving tasks comfortably. |  |  |  |  | **0.576** |
| OJFQ **36** My ability to tolerate crises greatly influences my stress levels at work |  |  |  |  | **0.494** |
| OJFQ **34** Lacking of expert in my field causing me stress |  |  |  |  | **0.456** |

**Table 7: Reliability of the OJFQ (N=139)**

| **Components** | **No. of Items** | **Mean** | **SD** | **Cronbach’s Alpha** | **Correlation**  **between 5 factors and total score^a^** | **Composite Reliability (CR)** | **Interpretation** |
| --- | --- | --- | --- | --- | --- | --- | --- |
| **Total score of the questionnaire** | **37** | **179.05** | **22.096** | **0.774** |  |  |  |
| **Factor 1: Management and role clarity** | **10** | **48.64** | **9.781** | **0.820** | **.825**** | **0.8337** | **Good** |
| **Factor 2: Emotional regulation and coping responses** | **8** | **20.95** | **4.688** | **0.821** | **.183*** | **0.8333** | **Good** |
| **Factor 3: Career development and engagement** | **8** | **22.21** | **7.161** | **0.811** | **.437**** | **0.8468** | **Excellent** |
| **Factor 4: Workplace bureaucracy and social challenges** | **7** | **36.10** | **6.793** | **0.785** | **.635**** | **0.7630** | **Acceptable** |
| **Factor 5: Workplace resources and stability** | **4** | **16.82** | **3.224** | **0.672** | **.347**** | **0.6755** | **Moderate** |

**^a^ Spearman’s rank correlation coefficient, **p<0.01.**

**Table 8. Construct validity of the OJFQ**

| **Five-Factor Model** | **X2/df** | **GFI** | **AGFI** | **NNFI** | **NFI** | **CFI** | **IFI** | **RFI** | **PNFI** | **PGFI** |
| --- | --- | --- | --- | --- | --- | --- | --- | --- | --- | --- |
| **Scale** | 1199/619=1.9 | 0.689 | 0.647 | 0.651 | 0.511 | 0.675 | 0.684 | 0.474 | 0.475 | 0.607 |

X2 = Chi-square, df = degrees of freedom. GFI = Goodness of fit index, AGFI = Adjusted goodness of fit index, NNFI = Non-normed fit

index, NFI = Normed fit index, CFI = Comparative fit index, IFI = Incremental fit index, RFI = Relative fit index, PNFI = Parsimony normed

fit index, PGFI = Parsimony goodness of fit index.

**Fig 1. Integrated Conceptual Framework of Development of Job Frustration**

_Ability to Perform Task_

_Motivation_

_Role Perception_

**_(Co-Variable)_**

_Flight the situation_

_Initial Alarm Reaction_

_Flight from the situation_

_Resistance_

_Failure to Fight/ Lower the impact of stress_

_Adapts with the situation_

_Emotional exhaustion_

_MARS Model_

_Situational Factors (demographic,_ social, leadership, work culture)

_Compromise_

_Aggression_

_Autonomy (Self-efficacy)_

_Competence_

**_(Co-Variable)_**

_Desi’s Self Determination Theory_

_Relatedness_

_Rationalization_

_Withdrawal_

_Job Frustration_

_Failure Provokes_

_Fixation_

_General Adaptation Syndrome_

_Layers of Frustration_

_Path to_

Personal-related

Work-related

Client-Related

Copenhagen Burnout Inventory

**Fig 2. Scree Plot Performed On 5-Factors with Eigenvalue Greater Than 1**


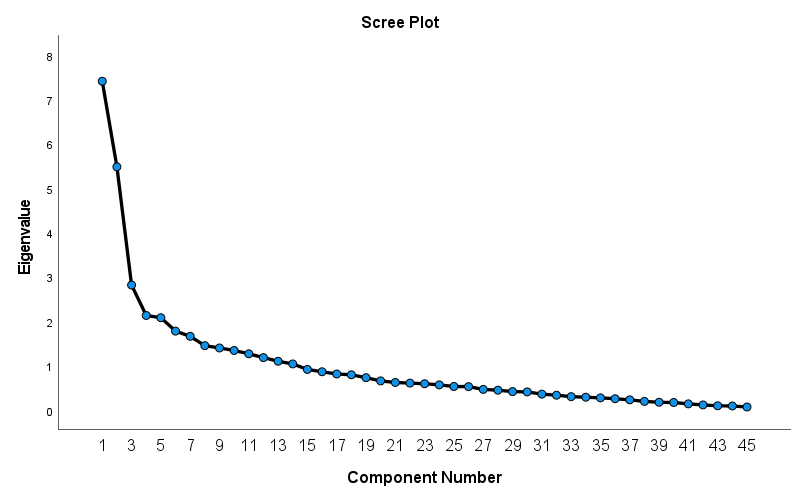


**Fig 3. Confirmatory Factor Analysis Model of the OJFQ**


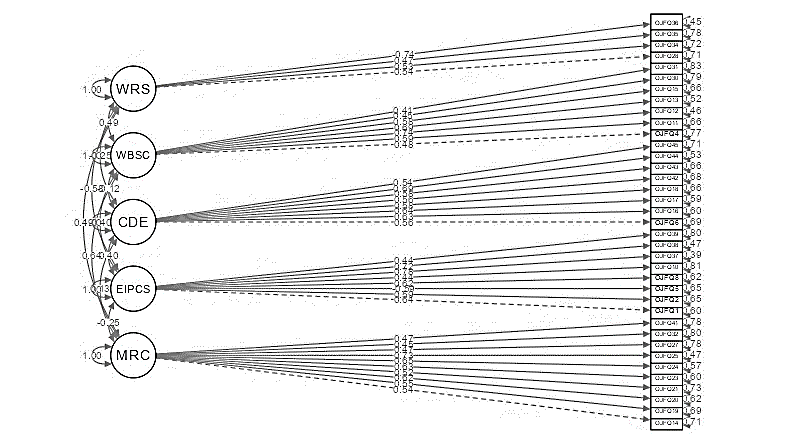


**Fig. 4. Scatter Plot of OJFQ and CBI Scores**


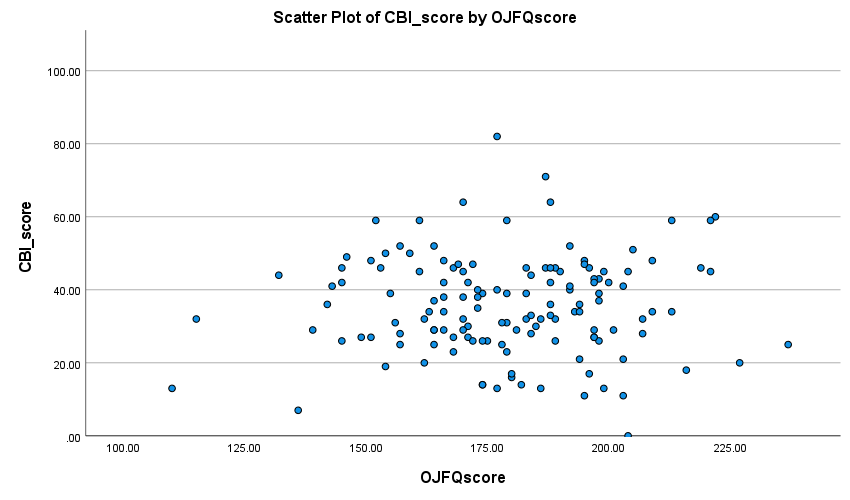

Supplement: Supplementary file 1 — Supplementary Material 1. [file 40359_2026_4762_MOESM1_ESM.docx]
