# Supplementary material for: Measuring job frustration in Omani healthcare workers: development and psychometric validation of the OJFQ
Source: BMC Psychol. 2026 May 19;14:1029. doi: 10.1186/s40359-026-04762-5 (PMC13352741; doi:10.1186/s40359-026-04762-5)

**Table 1. Demographic characteristics of the participants (N=140)**

| Item | N (%) | Mean |  | SD |
| --- | --- | --- | --- | --- |
| **Age (years)** | - | 33.18 | + | 6.207 |
| **Work experience (years)** | - | 9.72 | + | 5.803 |
| **Gender** |  |  |  |  |
| Male | 67 (47.5%) |  |  |  |
| Female | 73 (51.8%) |  |  |  |
| **Field of practice** |  |  |  |  |
| Clinical | 61 (43.3%) |  |  |  |
| Leadership | 77 (54.6%) |  |  |  |

**Table 2. Item analysis of OJFQ (N= 140)**

| **Items** | **Mean** | **SD** | **Skewness** | | **Kurtosis** | | **t** | **95% CI** | | **Correlation with total score** |
| --- | --- | --- | --- | --- | --- | --- | --- | --- | --- | --- |
|  |  |  | **Statistic** | **Std. Error** | **Statistic** | **Std. Error** |  | **Lower** | **Upper** |  |
| **Total Score of Scale** | **179.0504** | **22.09608** | **-.222-** | **.206** | **.322** | **.408** | **95.536** | **175.3446** | **182.7562** | **1** |
| **Q1** | **2.17** | **1.258** | **1.387** | **.205** | **2.114** | **.407** | **20.428** | **1.96** | **2.38** | **.168*** |
| **Q2** | **2.09** | **1.042** | **1.297** | **.205** | **1.158** | **.407** | **23.680** | **1.91** | **2.26** | **.238**** |
| **Q3** | **2.71** | **1.407** | **1.055** | **.205** | **.810** | **.407** | **22.773** | **2.47** | **2.94** | **.280**** |
| **Q4** | **5.33** | **1.476** | **-1.020-** | **.205** | **.489** | **.407** | **24.711** | **5.08** | **5.58** | **.411**** |
| **Q5** | **5.70** | **1.216** | **-1.525-** | **.205** | **2.808** | **.407** | **55.481** | **5.50** | **5.90** | **.107** |
| **Q6** | **3.16** | **1.576** | **.966** | **.205** | **.018** | **.407** | **23.757** | **2.90** | **3.43** | **.198*** |
| **Q7** | **4.83** | **1.799** | **-.942-** | **.205** | **-.241-** | **.407** | **31.757** | **4.53** | **5.13** | **.136** |
| **Q8** | **2.06** | **1.051** | **1.431** | **.205** | **3.084** | **.407** | **23.159** | **1.88** | **2.23** | **.110** |
| **Q9** | **5.62** | **1.354** | **-1.293-** | **.205** | **1.299** | **.407** | **49.114** | **5.40** | **5.85** | **.182*** |
| **Q10** | **2.46** | **1.305** | **1.110** | **.205** | **.645** | **.407** | **22.277** | **2.24** | **2.68** | **-.036-** |
| **Q11** | **5.13** | **1.559** | **-.980-** | **.205** | **.162** | **.407** | **38.932** | **4.87** | **5.39** | **.322**** |
| **Q12** | **5.36** | **1.425** | **-1.075-** | **.205** | **.841** | **.407** | **44.528** | **5.13** | **5.60** | **.450**** |
| **Q13** | **4.86** | **1.532** | **-.669-** | **.205** | **-.263-** | **.407** | **37.558** | **4.61** | **5.12** | **.583**** |
| **Q14** | **4.92** | **1.532** | **-.622-** | **.205** | **-.279-** | **.407** | **38.016** | **4.67** | **5.18** | **.513**** |
| **Q15** | **5.24** | **1.478** | **-.984-** | **.205** | **.355** | **.407** | **41.965** | **5.00** | **5.49** | **.267**** |
| **Q16** | **3.02** | **1.620** | **.963** | **.205** | **-.041-** | **.407** | **22.064** | **2.75** | **3.29** | **.490**** |
| **Q17** | **2.38** | **1.135** | **1.160** | **.205** | **1.135** | **.407** | **24.805** | **2.19** | **2.57** | **.502**** |
| **Q18** | **2.86** | **1.447** | **.773** | **.205** | **-.296-** | **.407** | **23.359** | **2.62** | **3.10** | **.656**** |
| **Q19** | **4.44** | **1.855** | **-.391-** | **.205** | **-1.141-** | **.407** | **28.290** | **4.13** | **4.75** | **.649**** |
| **Q20** | **4.64** | **1.734** | **-.450-** | **.205** | **-.853-** | **.407** | **31.673** | **4.35** | **4.93** | **.495**** |
| **Q21** | **4.58** | **1.650** | **-.398-** | **.205** | **-.869-** | **.407** | **32.741** | **4.31** | **4.86** | **.257**** |
| **Q22** | **4.28** | **1.957** | **-.252-** | **.205** | **-1.260-** | **.407** | **25.874** | **3.95** | **4.61** | **.519**** |
| **Q23** | **5.10** | **1.416** | **-.581-** | **.205** | **-.206** | **.407** | **42.624** | **4.86** | **5.34** | **.460**** |
| **Q24** | **5.06** | **1.425** | **-.568-** | **.205** | **-.187-** | **.407** | **42.038** | **4.83** | **5.30** | **.364**** |
| **Q25** | **4.89** | **1.455** | **-.638-** | **.205** | **-.218-** | **.407** | **39.737** | **4.64** | **5.13** | **.333**** |
| **Q26** | **4.79** | **1.492** | **-.482-** | **.205** | **-.476** | **.407** | **37.944** | **4.54** | **5.04** | **.392**** |
| **Q27** | **4.26** | **1.922** | **-.229-** | **.205** | **-1.306-** | **.407** | **26.258** | **3.94** | **4.59** | **.320**** |
| **Q28** | **5.05** | **1.538** | **-.832-** | **.205** | **-.004-** | **.407** | **38.859** | **4.79** | **5.31** | **.225**** |
| **Q29** | **4.71** | **1.557** | **-.486-** | **.205** | **-.535-** | **.407** | **35.770** | **4.45** | **4.97** | **-.135-** |
| **Q30** | **4.91** | **1.586** | **-.679-** | **.205** | **-.443-** | **.407** | **36.601** | **4.64** | **5.17** | **.310**** |
| **Q31** | **5.26** | **1.506** | **-.858-** | **.205** | **-.131-** | **.407** | **41.362** | **5.01** | **5.52** | **.295**** |
| **Q32** | **5.67** | **1.375** | **-1.122-** | **.205** | **.917** | **.407** | **48.796** | **5.44** | **5.90** | **.268**** |
| **Q33** | **5.48** | **1.486** | **-1.132-** | **.205** | **.515** | **.407** | **43.623** | **5.23** | **5.73** | **.384**** |
| **Q34** | **4.89** | **1.539** | **-.718** | **.205** | **-.468-** | **.407** | **37.608** | **4.64** | **5.15** | **.299**** |
| **Q35** | **4.23** | **1.715** | **-.223-** | **.205** | **-1.005-** | **.407** | **29.179** | **3.94** | **4.52** | **.266**** |
| **Q36** | **2.65** | **1.175** | **1.172** | **.205** | **1.234** | **.407** | **26.689** | **2.45** | **2.85** | **.582**** |
| **Q37** | **2.21** | **.973** | **.983** | **.205** | **1.119** | **.407** | **26.929** | **2.05** | **2.38** | **.583**** |
| **Q38** | **2.31** | **1.150** | **1.361** | **.205** | **2.070** | **.407** | **23.740** | **2.11** | **2.50** | **.515**** |
| **Q39** | **1.96** | **1.024** | **1.473** | **.205** | **2.602** | **.407** | **22.615** | **1.79** | **2.13** | **.636**** |
| **Q40** | **1.96** | **1.045** | **1.507** | **.205** | **2.599** | **.407** | **22.164** | **1.78** | **2.13** | **.027** |
| **Q41** | **5.04** | **1.416** | **-.680-** | **.205** | **-.104-** | **.407** | **42.070** | **4.80** | **5.27** | **.119** |
| **Q42** | **2.94** | **1.358** | **.974** | **.205** | **.617** | **.407** | **25.575** | **2.71** | **3.16** | **.034** |
| **Q43** | **2.70** | **1.262** | **1.173** | **.205** | **1.295** | **.407** | **25.313** | **2.49** | **2.91** | **.087** |
| **Q44** | **2.81** | **1.267** | **.787** | **.205** | **-.034-** | **.407** | **26.280** | **2.60** | **3.03** | **.288**** |
| **Q45** | **2.34** | **1.174** | **1.197** | **.205** | **1.662** | **.407** | **23.615** | **2.15** | **2.54** | **.285**** |

****. Correlation is significant at the 0.01 level (2-tailed).**

***. Correlation is significant at the 0.05 level (2-tailed).**

**Table 3. Rotated factors for principle component analysis of OJFQ**

| **Items** | **Factor Loading** | | | | |
| --- | --- | --- | --- | --- | --- |
|  | **I** | **II** | **III** | **IV** | **V** |
| **Factor 1: Management and role clarity** |  |  |  |  |  |
| OJFQ **24** There is a waste of effort in succession planning since the selection of new healthcare leaders comes solely from top authorities. | 0.745 |  |  |  |  |
| OJFQ **25** My concerns are acknowledged by the top management, but no substantial actions are taken to address them. | 0.713 |  |  |  |  |
| OJFQ **23** There is extreme interference from top management in leaders’ tasks. | 0.702 |  |  |  |  |
| OJFQ **41** It is hard to change basic policies in the hospital to adapt to the dynamicity of technological advances | 0.578 |  |  |  |  |
| OJFQ **19** I am often blamed when a clinical-related adverse incident occurs | 0.570 |  |  |  |  |
| OJFQ **32** Working on shift duties is affecting my social life | 0.521 |  |  |  |  |
| OJFQ **21** Shift supervisors are reluctant to make clinical decisions without consulting their immediate manager | 0.493 |  |  |  |  |
| OJFQ**14** There is unclear delineation of roles among different members of the healthcare multidisciplinary team | 0.477 |  |  |  |  |
| OJFQ**20** If I accept extra work responsibilities, I will be blamed for the extra work not being done as required | 0.476 |  |  |  |  |
| OJFQ**27** I as a healthcare worker prefer to remain silent at work | 0.474 |  |  |  |  |
| **Factor 2: Emotional intelligence and professional coping strategies** |  |  |  |  |  |
| OJFQ **8** My level of commitment to task is enhanced when I involve in decision-making |  | **0.751** |  |  |  |
| OJFQ **37** My resilience at work improves when I can effectively understand and manage emotions |  | **0.712** |  |  |  |
| OJFQ1 My emotional well-being significantly improves when a manager demonstrates empathy. |  | **0.684** |  |  |  |
| OJFQ **5** I am stressed when there is lack of teamwork |  | **0.679** |  |  |  |
| OJFQ **38** My ability to recognize emotions in myself and others improves my coping skills |  | **0.618** |  |  |  |
| OJFQ **2** My understanding of the stressors enhances my affective state |  | **0.577** |  |  |  |
| OJFQ **10** I feel less frustrated when the workload is distributed fairly in my workplace. |  | **0.510** |  |  |  |
| OJFQ **39** My faith or religious beliefs provide me with strength to cope with work-related stress |  | **0.400** |  |  |  |
| **Factor 3: Career development and engagement** |  |  |  |  |  |
| OJFQ **44** My training materials are directly applicable to my daily tasks that reduce my job frustration |  |  | **0.735** |  |  |
| OJFQ **43** I am actively participating in own learning process to reduce my job frustration |  |  | **0.730** |  |  |
| OJFQ **42** I receive constructive feedback that help improving my skills, performance and learning that lead to less job frustration |  |  | **0.705** |  |  |
| OJFQ **16** My current working environment is safe for me to practice new skills |  |  | **0.674** |  |  |
| OJFQ **45** I feel that staying updated with the latest trends in healthcare helps me manage my job frustration |  |  | **0.631** |  |  |
| OJFQ **17** I am having a supportive relationship with my leader that reduce my job frustration and improves my performance |  |  | **0.588** |  |  |
| OJFQ **18** I am getting positive reinforcement and encouragement at work like everyone else |  |  | **0.530** |  |  |
| OJFQ **6** My performance improves with the presence of supervisors. |  |  | **0.503** |  |  |
| **Factor 4: Workplace bureaucracy and social challenges** |  |  |  |  |  |
| OJFQ **12** I feel overwhelmed doing additional administrative responsibilities under unhelpful management policies. |  |  |  | **0.674** |  |
| OJFQ **30** There is gender stereotype in healthcare related fields that stressed me up |  |  |  | **0.639** |  |
| OJFQ **11** My frustration caused by unclear/ lack of policies and procedures |  |  |  | **0.598** |  |
| OJFQ **13** I am frustrated by the demanding administration works |  |  |  | **0.547** |  |
| OJFQ **31** I need to put more efforts to balance between professional and personal life |  |  |  | **0.500** |  |
| OJFQ **15** I feel lack of confidence due to poorly defined job descriptions and task allocations among different members of the healthcare multidisciplinary team |  |  |  | **0.495** |  |
| OJFQ **4** I feel that congested offices with a lack of privacy are not conducive to achieving tasks comfortably. |  |  |  | **0.414** |  |
| **Factor 5: Workplace resources and stability** |  |  |  |  |  |
| OJFQ **35** Worries about losing my job make me less satisfied with my current position |  |  |  |  | **0.794** |
| OJFQ **28** I feel that congested offices with a lack of privacy are not conducive to achieving tasks comfortably. |  |  |  |  | **0.576** |
| OJFQ **36** My ability to tolerate crises greatly influences my stress levels at work |  |  |  |  | **0.494** |
| OJFQ **34** Lacking of expert in my field causing me stress |  |  |  |  | **0.456** |

**Table 4: Reliability of the OJFQ (N=140)**

| **Components** | **Mean** | **SD** | **Cronbach’s Alpha** | **Correlation**  **between 5 factors and total score^a^** |
| --- | --- | --- | --- | --- |
| **Total score of the questionnaire** | **179.05** | **22.096** | **0.774** |  |
| **Factor 1: Management and role clarity** | **48.64** | **9.781** | **0.820** | **.825**** |
| **Factor 2: Emotional intelligence and professional coping strategies** | **20.95** | **4.688** | **0.821** | **.183*** |
| **Factor 3: Career development and engagement** | **22.21** | **7.161** | **0.811** | **.437**** |
| **Factor 4: Workplace bureaucracy and social challenges** | **36.10** | **6.793** | **0.785** | **.635**** |
| **Factor 5: Workplace resources and stability** | **16.82** | **3.224** | **0.672** | **.347**** |

**^a^ Spearman’s rank correlation coefficient, **p<0.01.**

**Table 5. Construct validity of the OJFQ**

| **Five-Factor Model** | **X2/df** | **GFI** | **AGFI** | **NNFI** | **NFI** | **CFI** | **IFI** | **RFI** | **PNFI** | **PGFI** |
| --- | --- | --- | --- | --- | --- | --- | --- | --- | --- | --- |
| **Scale** | 1199/619=1.9 | 0.689 | 0.647 | 0.651 | 0.511 | 0.675 | 0.684 | 0.474 | 0.475 | 0.607 |

X2 = Chi-square, df = degrees of freedom. GFI = Goodness of fit index, AGFI = Adjusted goodness of fit index, NNFI = Non-normed fit

index, NFI = Normed fit index, CFI = Comparative fit index, IFI = Incremental fit index, RFI = Relative fit index, PNFI = Parsimony normed

fit index, PGFI = Parsimony goodness of fit index.

**Fig 1. Confirmatory Factor Analysis Model of the OJFQ**


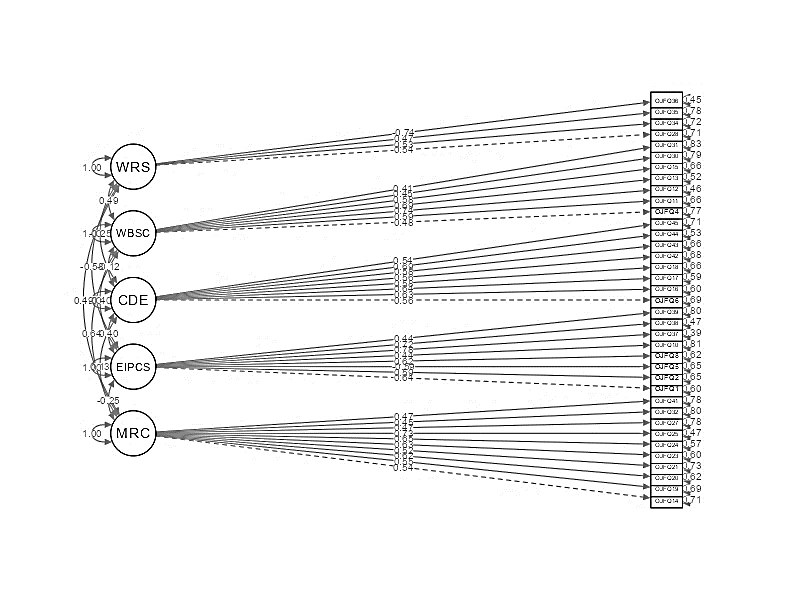

Supplement: Supplementary file 2 — Supplementary Material 2. [file 40359_2026_4762_MOESM2_ESM.docx]
